# Computational and biological profile of boronic acids for the detection of bacterial serine- and metallo-β-lactamases

**DOI:** 10.1038/s41598-017-17399-7

**Published:** 2017-12-18

**Authors:** Matteo Santucci, Francesca Spyrakis, Simon Cross, Antonio Quotadamo, Davide Farina, Donatella Tondi, Filomena De Luca, Jean-Denis Docquier, Ana Isabel Prieto, Claudia Ibacache, Jesús Blázquez, Alberto Venturelli, Gabriele Cruciani, Maria Paola Costi

**Affiliations:** 1 Department of Life Sciences, University of Modena and Reggio Emilia, Via Campi 103, 41125 Modena, Italy; 2 Molecular Discovery Limited, 215 Marsh Road, Pinner Middlesex, London, HA5-5NE United Kingdom; 3 TYDOCK PHARMA S.r.l., Strada Gherbella 294/b, Modena, 41126 Italy; 4 Dipartimento di Biotecnologie Mediche, University of Siena, Viale Bracci 16, 53100 Siena, Italy; 5 Biomedicine Institute of Sevilla (IBIS)-CSIC, Avda. Manuel Siurot, sn., Sevilla, Spain; 6 National Center of Biotechnology-CSIC, Calle Darwin, 3, 28049 Madrid, Spain; 7 Department of Chemistry, Biology and Biotechnology, University of Perugia, Via Elce di Sotto 8, 06123 Perugia, Italy; 8 Present Address: Department of Drug Science and Technology, University of Turin, Via Pietro Giuria 9, 10125 Turin, Italy

## Abstract

β-Lactamases (BLs) able to hydrolyze β-lactam antibiotics and more importantly the last resort carbapenems, represent a major mechanism of resistance in Gram-negative bacteria showing multi-drug or extensively drug resistant phenotypes. The early detection of BLs responsible of resistant infections is challenging: approaches aiming at the identification of new BLs inhibitors (BLI) can thus serve as the basis for the development of highly needed diagnostic tools. Starting from benzo-[b]-thiophene-2-boronic acid (BZB), a nanomolar inhibitor of AmpC β-lactamase (*K*_*i*_ = 27 nM), we have identified and characterized a set of BZB analogues able to inhibit clinically-relevant β-lactamases, including AmpC, Extended-Spectrum BLs (ESBL), KPC- and OXA-type carbapenemases and metallo-β-lactamases (MBL). A multiligand set of boronic acid (BA) β-lactamase inhibitors was obtained using covalent molecular modeling, synthetic chemistry, enzyme kinetics and antibacterial susceptibility testing. Data confirmed the possibility to discriminate between clinically-relevant β-lactamases on the basis of their inhibition profile. Interestingly, this work also allowed the identification of potent KPC-2 and NDM-1 inhibitors able to potentiate the activity of cefotaxime (CTX) and ceftazidime (CAZ) against resistant clinical isolates (MIC reduction, 32-fold). Our results open the way to the potential use of our set of compounds as a diagnostic tool for the sensitive detection of clinically-relevant β-lactamases.

## Introduction

Antibiotic resistance is a serious concern in hospital and community settings. Production of β-lactamases (BLs) is a prevalent resistance mechanism in bacterial infections and a threat to the available antibiotic armamentarium^1–3^. Novel BLs inhibitors are needed as well as a rapid clinical detection of BLs: the early recognition of resistant organisms can guide the assessment of the best treatment.

Nowadays more than 1,300 unique β-lactamases have been identified^4^, comprising serine BLs (classes A, C, D)^5^ and metallo BLs (MBLs, class B)^6^. The emergence, in particular, of KPC-type enzymes resistant to colistin^7–9^, CTX-M type associated with resistance to quinolones and aminoglycosides^10^ and with *bla*NDM-1-carrying plasmids^11^, AmpC enzymes of which overproduction is coupled with other resistant mechanism (efflux pumps and OprD porin loss)^12^ and VIM- and NDM-type associated with multiple resistance determinants on narrow and broad host-range plasmids^13^ led to the diffusion of multi-drug resistant (MDR) pathogens, and the emergence of extensively drug or pan-drug resistant isolates, significantly narrowing the antibiotic treatment options^14,15^.

In the last years, several new β-lactamase inhibitors entered clinical trials or received FDA-approval, including diazabicyclooctanes (avibactam, relebactam and nacubactam) and the boronic acid vaborbactam (RPX7009). The latter class of BL inhibitors covalently and reversibly inhibit serine-BLs and revert β-lactam resistance in BL-producing bacteria. The new antimicrobial combination with meropenem and the boronic acid BL inhibitor vaborbactam received FDA approval in August 2017^16^. Some boronic acids were also shown to inhibit the metallo-β-lactamases (MBLs), thus opening the way for the development of pan-β-lactamase inhibitors^17^. Boronic acids possess high potentiality for the development of useful therapeutics^18–23^ but also represent a valid tool for the early phenotypic detection of BLs produced by clinical isolates. Boronic acids inhibitors are already used for BLs detection in phenotypic tests (agar-diffusion combo tests)^24–26^ with other methods such as microarray profiles DNA detection^27^, chromogenic tests^28^ and double-disk diffusion test^29,30^.

However, if phenyl-boronic acid (PBA) and its analog 3-amino-phenyl-boronic acid (APBA) have been reported for diagnostic application in hospital setting^31,32^, the method has critical limits: boronic acids tend to bind saccharides and react unselectively with proteases^33,34^. Thus, the use of boronic acids in diagnostic tests useful for BLs detection in clinical samples show an important margin for improvement. Our approach relies on the substitution of a single boronic acid reagent by a set of ligands with fine-tuned properties to allow a more sensitive and informative BL detection test to be implemented. The ability of single compounds in a multi-ligand set to inhibit specific BLs with different potencies would potentially allow to discriminate between various types of clinically-relevant BLs produced by a clinical isolate *via* the analysis of the resulting inhibition profile.

In this context, we report the characterization of a set of benzo[b]thiophene-2-boronic acid derivatives showing differentiated inhibition profiles towards clinically relevant BLs. Selected boronic acids were then investigated for their potential application in phenotypic tests for the simple and sensitive detection of the production of various β-lactamases in clinical isolates^24,25^.

The multi-ligands set concept has a valuable diagnostic application. In this regard, a panel of clinically-relevant BLs was selected for this study and include CTX-M-15 ESBL, the KPC-2 and OXA-24 carbapenemases, AmpC-type enzymes and the NDM-1 and VIM-2 MBLs.

**Figure 1:**
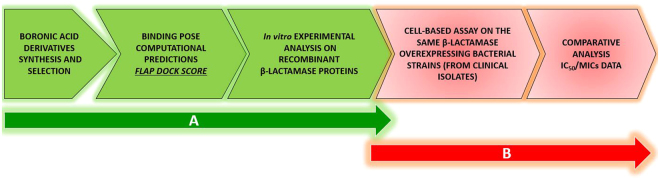
Project Work flow.

For the characterization and validation of a multi-ligand set, we followed a two-step strategy. First, an inhibitor-ligand discovery approach integrated with organic chemistry, covalent modeling and enzymatic tests allowed the selection of compounds with distinctive inhibition profile. An *ad hoc* modified version of FLAPdock algorithm implemented in the software FLAP and based on GRID Molecular Interaction Fields (MIFs)^35–37^ was used to characterize the covalent binding profile of the compound set against serine-BLs and the non-covalent binding towards metallo-BLs. The *in silico* inhibition profile obtained for each compound was confirmed *in vitro* with enzyme assays. Second, these compounds were evaluated for their ability to act synergistically with various β-lactam antibiotics to validate their potential use in phenotypic BL detection tests. A set of clinical isolates producing various BLs was used to test the synergistic activity of selected compounds with the third-generation cephalosporins ceftazidime and cefotaxime (Fig. 1). Interestingly, acyclic boronic acid derivatives are for the first time reported to inhibit metallo-β-lactamases, mimicking the transition state analogues *via* a non-covalent binding mode.

The comparison of the enzyme inhibition profile of each compound with its synergistic activity in susceptibility assays allowed the final selection of compounds in the multi-ligand set.

## Results

### Selection and synthesis of benzo[b]thiophene-2-boronic acid derivatives

Starting from a previously reported boronic acids library active *vs* AmpC with nanomolar potency^22^ we selected a series of six BZB derivatives of compound 1 (Table 1a), decorated with substituents different for size and properties. The two positions of derivatization, *i.e*. C5 and C7, were selected according to AmpC BL binding site structural properties and to synthetic accessibility^38^. The C5 position was previously reported to primarily affect the pharmacokinetic rather than the affinity towards AmpC BL^22^. For the C5 serie in the present work we effectively optimized the chemical synthesis, designing a more suitable protocol (SI Scheme S1A)^22,39^. For C7 derivatives, in turn, three BZB derivatives were obtained *via* a new synthetic scheme by introducing bulkier substituent (compounds **4**,**5** and **6**) (SI Scheme S1B).

**Table 1:**
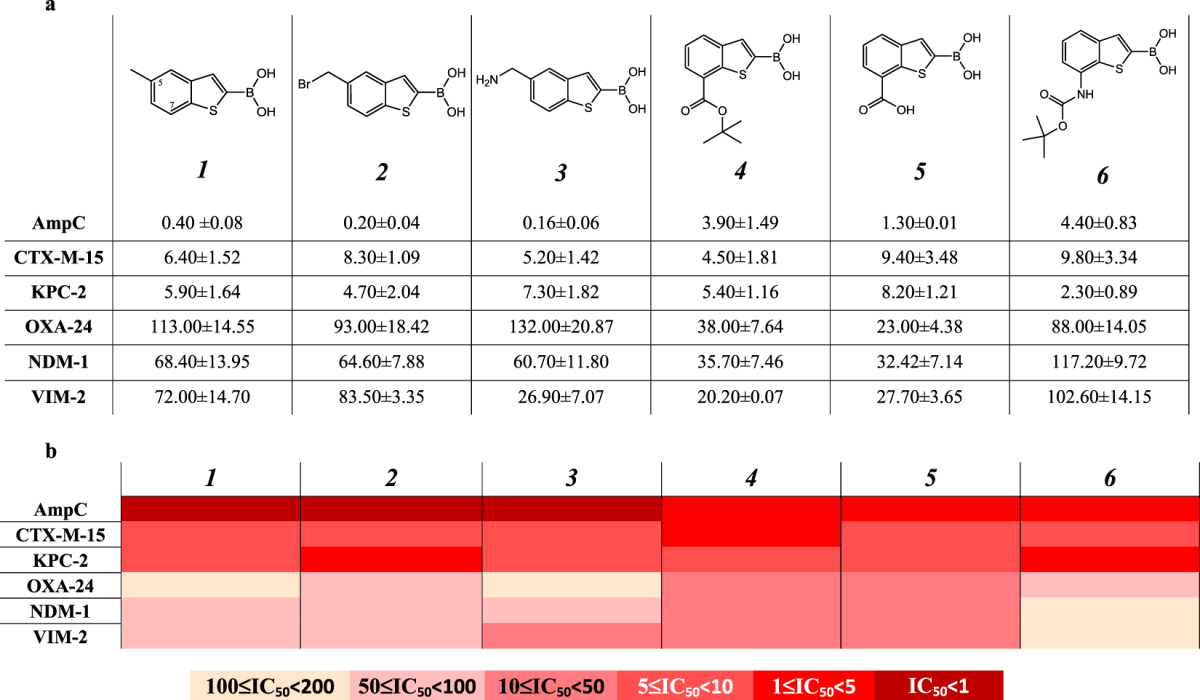
Chemical structure and IC_50_ (μM) values estimated for the compounds series towards the BLs panel: a - Compounds were prepared as pinacol-protected derivatives on the boronic group. The reported IC_50_ values are the mean values of two replicates for each tested inhibitor concentration value; the STDs were calculated by *Student’s t-test* considering a 95% of confidence range (ρ < 0.05); **b -** b. Heat-map representation of the above reported IC_50_ values.

### Inhibition profile of boronic acids towards clinically-relevant BLs

To obtain a well-characterized inhibition profile for each selected boronic acids against each BLs in the panel, the compounds were tested for inhibitory activity. The protein panel included clinically- and epidemiologically-relevant β-lactamases, representatives of each structural class (A to D), as mentioned above. Each BL was overexpressed in *E. coli*, purified by chromatography and characterized as already reported. (SI, Table S1)^40–45^. IC_50_ values were determined by measuring the initial rate of hydrolysis in the presence of five different inhibitor concentrations, in two independent replicates (ρ < 0.05) (see Materials and Methods section) (Table 1a,b).

Among serine-BLs, the tested compounds showed the strongest inhibition against AmpC, while the weakest inhibition was observed for the class D carbapenemase OXA-24. Compounds **1-3** present a different profile with respect to compounds **4-6**, being ten times more active against AmpC. In particular, compound **3** showed highest potency, with an IC_50_ of 0.16 µM. Compounds **4**, **5** and **6** show activity against AmpC, with low micromolar affinity. These variations could be reasonably attributed to the different structural modifications introduced at C5 (for **1**, **2** and **4)** and at C7 in compounds **4**, **5**, **6**, with the latters being significantly bulkier. The inhibition profiles versus CTX-M-15 and KPC-2 showed low micromolar IC_50_, *i.e*. 5–10 µM and 2–8 µM respectively (Table 1a,b)^39^. All compounds show lower affinity against OXA-24 with respect to the other BLs. The most promising derivatives, compounds **4** and **5**, present IC_50_ of 38 and 23 µM, respectively, while the others range from 88 µM up to 132 µM. Interesting results were obtained with compounds **4** and **5**, being active on the VIM-2 and NDM-1 MBLs, while maintaining inhibition of serine-BLs. Compound **6** presents an interesting inhibition profile *vs* the MBLs in the panel, with IC_50_ of 36 and 32 µM for NDM-1 and of 20 and 28 µM for VIM-2 respectively. Compound **3** was rather active on VIM-2 (IC_50_, 27 µM) but less on NDM-1. All other compounds were weaker inhibitors of MBLs with IC_50_ values ranging between 60 µM and 100 µM.

### Modeling boronic acid derivatives interactions within serine BLs binding site through covalent docking

To explain the inhibition profile observed *in vitro*, *in silico* BAs-BLs interaction studies were performed. Boronic acids act as transition-state analogue inhibitors^18^ interacting with the catalytic serine. The generated tetrahedral intermediate has similar geometry to that assumed by β-lactams when hydrolyzed by BL (Scheme S2). To investigate the role played by bonded and non–bonded interactions in BLs inhibition we modified the docking software FLAPdock to model the covalent binding of boronic acids to BLs binding site. Since the boronic acid core interacts with serine BLs exploiting the same covalent mechanism, the structural peculiarity of each BL binding site should account for the formation of additional non-bonded interactions and for the inhibition potency variability. The Molecular Interaction Fields (MIFs) for each selected BL binding sites were calculated (SI, Figure S1) representing the ligand image of each pocket, *i.e*. the structural and chemical requirements a ligand should fulfill to profitably interact with the protein. Therefore, the different MIFs depict how diverse are the binding sites of each protein, in terms of shape, hydrophobic and H-bond donor/acceptor hot spots. The FLAPdock predicted orientations of each compound within BLs binding sites are described below.

#### Inhibitors binding interaction with AmpC

**Figure 2:**
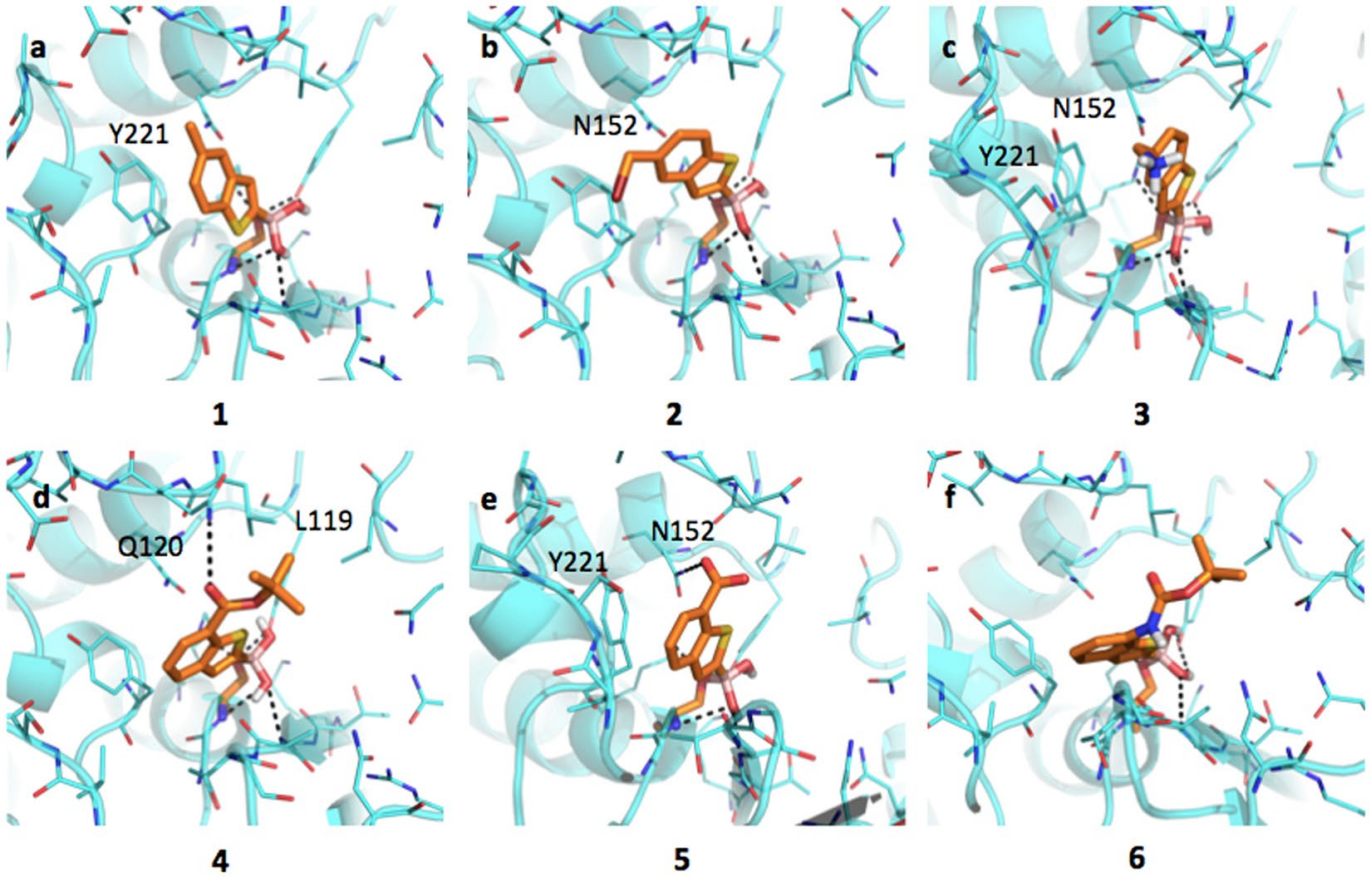
Docking poses for compounds in Table 1 in AmpC binding site (PDB code 1kdw). Residues lining the pocket are shown as capped sticks. Ligands are orange-coloured. Hydrogen bonds are represented by black dashed lines. Pictures were prepared using Pymol (http://www.pymol.org).

AmpC presents a rather large binding site, characterized by hydrophobic and H-bond donor regions (SI, Figure S1a). As highlighted by the docking simulations performed on AmpC from *P. aeruginosa* (Fig. 2), the boronic group of BAs interacts extensively with the surrounding residues. The whole acyl-enzyme complex is maintained in a deacetylating conformation^21,46^. The analysis of AmpC binding site reveals the presence of different hydrophobic regions, mainly in front of Tyr221. Planar compounds as **1**, **3** and occasionally **2** can form *pi-pi* interactions with this residue. Interestingly, these molecules can also assume an alternative orientation placing the benzene ring in front of Asn152, thus allowing the formation of a dipole-quadrupole contact, as seen in the X-ray structure of the AmpC-BZB complex (SI, Figure S2)^46^. Differently from **1**, compound **2** assumes an intermediate conformation (Fig. 2b), allowing the methyl-bromine to make hydrophobic contacts with Tyr221 and the benzene to form dipole-quadrupole interactions with Asn152. Compound **3** can still assume alternative conformations moving from Tyr221 to Asn152 side-chains (Fig. 2c). In general, the possibility of assuming different favorable conformations can increase the entropic free energy component upon ligand binding and, possibly, the protein-ligand stability. The loss of freedom, on the contrary, is associated to an entropic and free energy cost, opposing binding and favoring dissociation^47^. In its best-predicted orientation compound **4** forms hydrophobic interaction with Leu119 through the *tert*-butyl moiety and a H-bond with Gln120 (Fig. 2d). The bulkier side-chain could hinder ligand entrance and allocation, reduces the ligand mobility and prevents the formation of *pi-pi* contacts with Tyr221, thus decreasing the binding affinity (IC_50_ 3.88 μM). Compound **5** H-bond interacts with the carboxylic group and Asn152 side-chain and maintains the stacking interaction with Tyr221 (Fig. 2e). The presence of the carboxylic group affects the ligand orientation, forcing it to assume a more constrained pose. Compound **6** presents an orientation similar to **4** (Fig. 2f) maintaining the interaction with Asn152.

#### Inhibitors binding interaction with CTX-M-15

**Figure 3:**
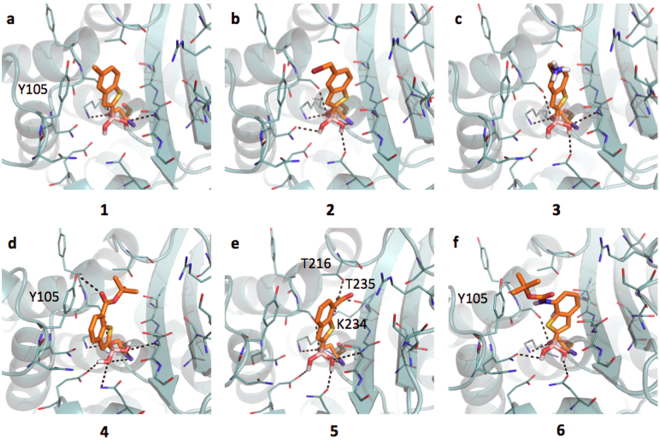
Docking poses for compounds in Table 1 in CTX-M-15 binding site (PDB code 4hbt). Residues lining the pocket are shown as capped sticks. Ligands are orange-coloured. Hydrogen bonds are represented by black dashed lines. Pictures were prepared using Pymol (http://www.pymol.org).

The active site of CTX-M-15 is notably different with respect to the previously described AmpC (SI, Figure S1b), while being more similar to that of KPC-2. CTX-M-15 has a large hydrophobic region in front of Tyr105, while the deeper portion of the pocket presents extended H-bond donor and acceptor areas. The boronic group, besides the covalent bond with the catalytic Ser70, is stabilized by H-bonds formed with the side chains of Lys73, Ser130, Asn132, Asn170, the backbone nitrogen of the same Ser70 and, sometimes, Ser237. BAs assume an acetylating conformation^21,48^. Compound **1** is well positioned in the pocket (Fig. 3a), with the methyl group pointing to a hydrophobic region of the site. Nevertheless, no *pi-pi* interaction is formed between the benzo(b)thiophene and the phenyl of Tyr105 (as Tyr221 in AmpC). This missing interaction, likely related to a different architecture of the binding site, might contribute to the affinity loss of **1** towards CTX-M-15 with respect to AmpC (IC_50_ 6.4 μM and 0.4 μM, respectively; 18 fold decrement) (Table 1a,b). Moreover, while in AmpC alternative orientations of the ligands were favorably assumed *via pi-pi* interaction with Tyr221 or dipole-quadrupole contacts with Asn152, in CTX-M-15 no residue similar to Asn152 is present thus no additional interactions are established. Compounds **2** and **3** (Fig. 3b,c) present similar orientation and affinity decrease with respect to AmpC. Compound **4** (Fig. 3d) properly orients its aromatic ring towards Tyr105 forming a *pi-pi* contact and occupying the main hydrophobic MIF of the binding site (IC_50_ 4.49 μM). Favorable contacts are also formed by **5**, which interacts through the carboxylic moiety with Thr216, Lys234 and Thr235 (Fig. 3e). Nevertheless, with respect to compounds **4** the favorable stacking with Tyr105 is lost (IC_50_ 9.4 μM). Less favorable appears the orientation of compound **6** within CTX-M-15 pocket (Fig. 3f). The *tert*-butyl moiety partially occupies the hydrophobic area in front of Tyr105 and the amidic nitrogen is possibly involved in a H-bond with Tyr105 aromatic ring (IC_50_ 9,80 μM, Table 1a,b)^49^.

#### Inhibitors binding interaction with KPC-2

**Figure 4:**
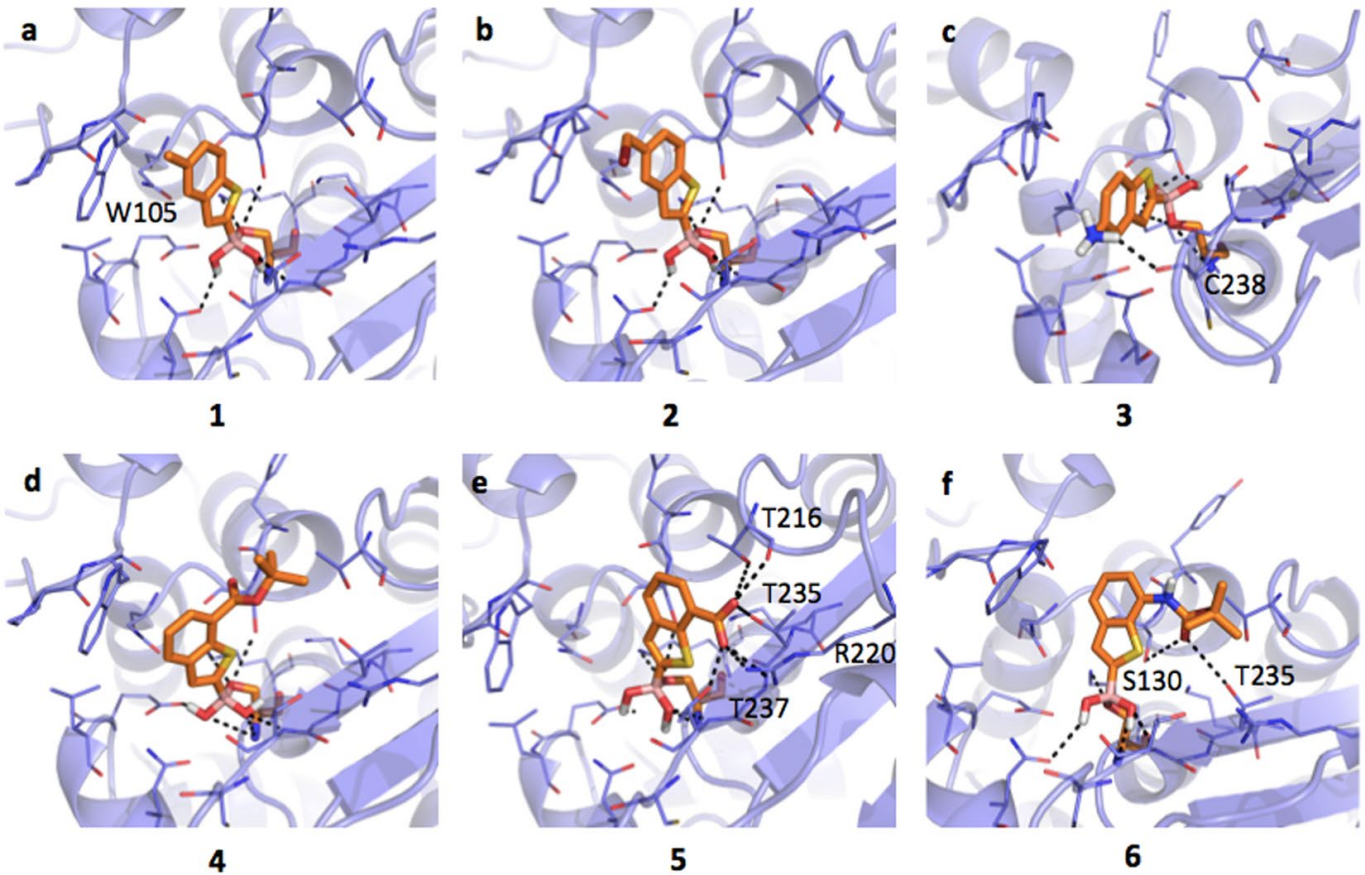
Docking poses for compounds in Table 1 in KPC-2 binding site (PDB code 3rxx). Residues lining the pocket are shown as capped sticks. Ligands are orange-coloured. Hydrogen bonds are represented by black dashed lines. Pictures were prepared using Pymol (http://www.pymol.org).

KPC-2 presents folding and pharmacophoric properties similar to CTX-M-15 (SI, Figure S1b, c). Trp105, like in CTX-M-15, plays a critical role in ligand binding and in the broad-spectrum activity of KPC-2 carbapenemases^50^. Again, the compounds are oriented in an acetylating conformation^21^. The boronic group is covalently bound to the catalytic Ser70 and is stabilized by H-bonds with Lys73, Ser130, Glu166, Asn170 and Thr237. Compounds **1**, **2** and **3** show similar orientations, with respect to CTX-M-15. Compound **1** locates the methyl moiety on the hydrophobic field generated by Trp105 and maintain similar affinity as in CTX-M-15 (Fig. 4a, IC_50_ 5.9 μM). Compound **2** locates the methyl bromine moiety in the same region (Fig. 4b, IC_50_ 4.7 μM). Compound **3** assumes two possible orientations, with the amino moiety interacting with the backbone carbonyl of Cys238 (Fig. 4c; IC_50_ 7.3 μM). No proper *pi-pi* contact is formed with Trp105. In general, the loss of stable hydrophobic interactions and the absence of Asn152, normally involved in dipole-quadrupole contacts with aromatic moieties, could be responsible for the lower affinity of compounds **1**, **2** and **3** towards class A BLs with respect to class C (Table 1a,b **)**. Compound **4** assumes an orientation similar to that in CTX-M-15 (Fig. 4d), while **5** interact extensively through the carboxylic moiety with residues Thr216, Arg220, Thr235 and Thr237 (Fig. 4e, IC_50_ 8.22 μM and 5.38 μM, respectively). H-bond contacts are as well undertaken by compound **6** carbonyl moiety with Ser130 and Thr235 side-chains (Fig. 4f; IC_50_ = 2.25 μM).

#### Inhibitors interaction with OXA-24

**Figure 5:**
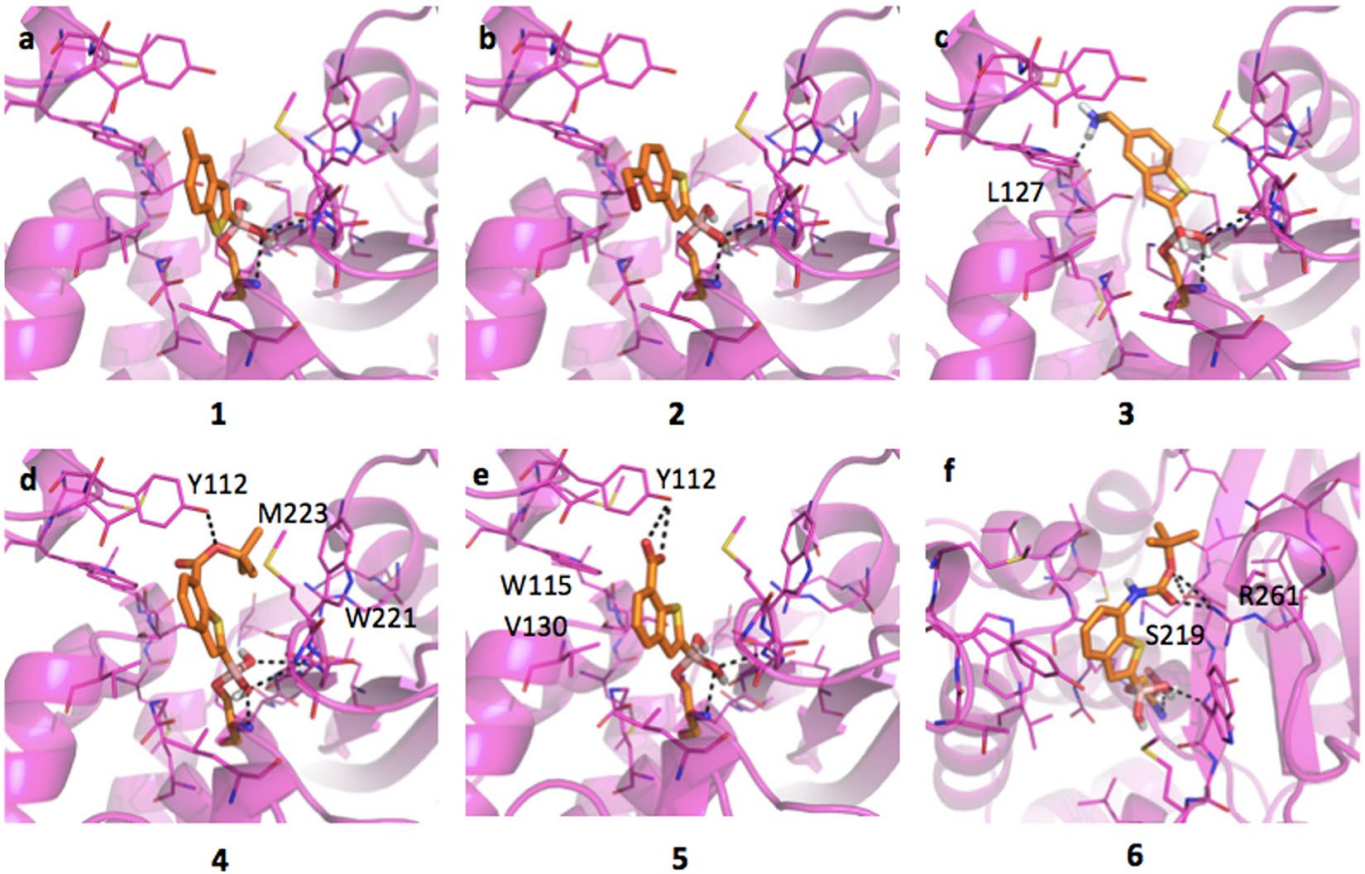
Docking poses for compounds in Table 1 in OXA-24 binding site (PDB code 3fv7). Residues lining the pocket are shown as capped sticks. Ligands are orange-coloured. Hydrogen bonds are represented by black dashed lines. Pictures were prepared using Pymol (http://www.pymol.org).

With respect to class A and C BLs, OXA-24 (class D), has a larger and more hydrophobic binding site (SI, Figure S1d), moreover no aromatic residues are properly oriented to form *pi-pi* contacts with the ligands. All compounds are stabilized by the formation of a covalent contact between the catalytic Ser81 and the boronic group, involved in further H-bonds with Ser81 and Trp221 backbone nitrogens. The acetylating orientation of CTX-M-15 and KPC-2 is maintained. Nevertheless, the boronic group, because of the different binding site architecture, undertakes fewer contacts. Compounds **1** and **2** assume similar orientations (Fig. 5a,b), fitting the hydrophobic contours, but not directly interacting with any residue, which turns in a poorer affinity (IC_50_ 113 μM and 93 μM, respectively). Compound **3** moves towards Leu127, to make a H-bond through the charged amino group, but away from the more hydrophobic region of the pocket (Fig. 5c; IC_50_ 132 μM). Higher affinity was observed for **4** and **5** (Figs 5d, 7e). Compound **4** shows a favorable orientation being able to H-bond to Tyr112 and to form hydrophobic interactions with Trp221 and Met223 *via* the *tert*-butyl group. Compound **5** is able to fit the hydrophobic contours provided by Trp115 and Val130 and to H-bond, through the carboxylic moiety, the hydroxyl group of Tyr112. Compound **6** assumes an orientation similar to **3** and forms H-bonds with Ser219 and Arg261, while hydrophobic interactions are lost (Fig. 5f; IC_50_ 88 μM). In general, the absence of relevant hydrophobic interactions and the lower number of contacts made by the boronic acid moiety might contribute to the loss of activity towards OXA-24, with respect to the previously analyzed BLs.

Through modeling we rationalized, for each ligand in the set, at the molecular level, the inhibition against the different serine BLs in the panel.

### Modeling boronic acid derivatives interactions within MBL binding site

 For the first time acyclic BAs are seen to extend their inhibitory affinity also vs MBLs, clearly suggesting that there is space to implement the affinity towards NDM- and VIM-type enzymes. For mechanistic reasons, the interaction of the boronic acids with NDM-1 and VIM-2 was modeled according to the mechanism of action reported by Brem *et al*. for cyclic boronic acids. This implies a nucleophilic attack by a hydroxide, bridging the two zinc ions in ground state structures, on the boron and the generation of a tetrahedral intermediate. The IC_50_ estimated for the compounds towards the two analyzed MBL are generally higher with respect to those observed for serine BL, with the exception of OXA-24. The lack of a covalent bond replaced by coordination interaction between the enzyme and the compounds likely explain this fall of inhibition activity.

### Inhibitors binding interaction with NDM-1

Both NDM-1 and VIM-2 present an overall similar fold and comparable hydrogen-bond donor and acceptor residues but different hydrophobic regions, the latter being quite larger in VIM-2 (SI, Figure S1e,f).

**Figure 6:**
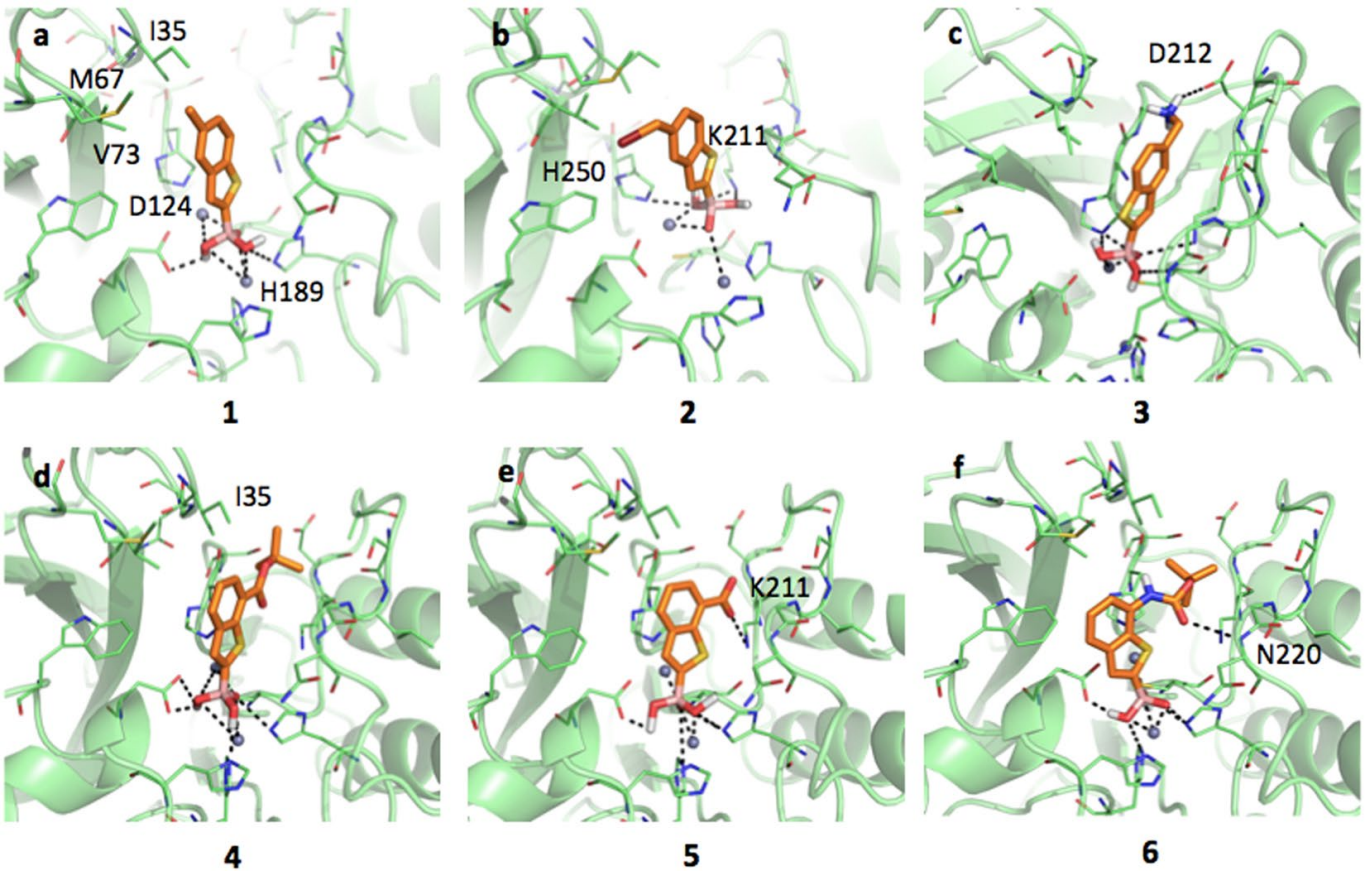
Docking poses for compounds in Table 1 in NDM-1 binding site (PDB code 3q6x). Residues lining the pocket are shown as capped sticks. Ligands are orange-coloured. Hydrogen bonds are represented by black dashed lines. Pictures were prepared using Pymol (http://www.pymol.org).

Compounds **1** and **2** have a very similar binding mode and inhibition potencies towards NDM-1 (68.40 μM and 64.60 μM, respectively). In our models, the boronic group was coordinating the two zinc ions, with H-bonds being formed between compound 1 and residues Asp124 and His189, while compound 2 interacts with residues Lys211 and His250 (Fig. 6a and b **)**. The methyl and methyl bromine moieties are located in a small hydrophobic patch delimited by Ile35, Met67 and Val73. Compound **3** forms a salt bridge interaction with Asp212, but moving in this direction loses any possible hydrophobic contact and the binding to one of the zinc ions. Accordingly, the inhibition potency slightly increases (IC_50_ equal to 60.70 μM; Fig. 6c). Compound **4**, **5** and **6** coordinates both metals in the same way as compounds **1** and **2**. Compound **4** (Fig. 6d) locates its *tert*-butyl moiety in a small hydrophobic region delimited by Ile35 but is not able to properly reach Lys211 that is, instead, salt bridged by compound **5** (Fig. 6e). Docking simulations suggested a possible alternative orientation for compound **5**, in which the carboxylic moiety directly coordinates one of the metals (not shown in the picture). Compound **6** is able to hydrogen bond to Asn220 and, differently from the two previous compounds, shows higher IC_50_ (102.60 μM; Fig. 6f).

### Inhibitors binding interaction with VIM-2

**Figure 7:**
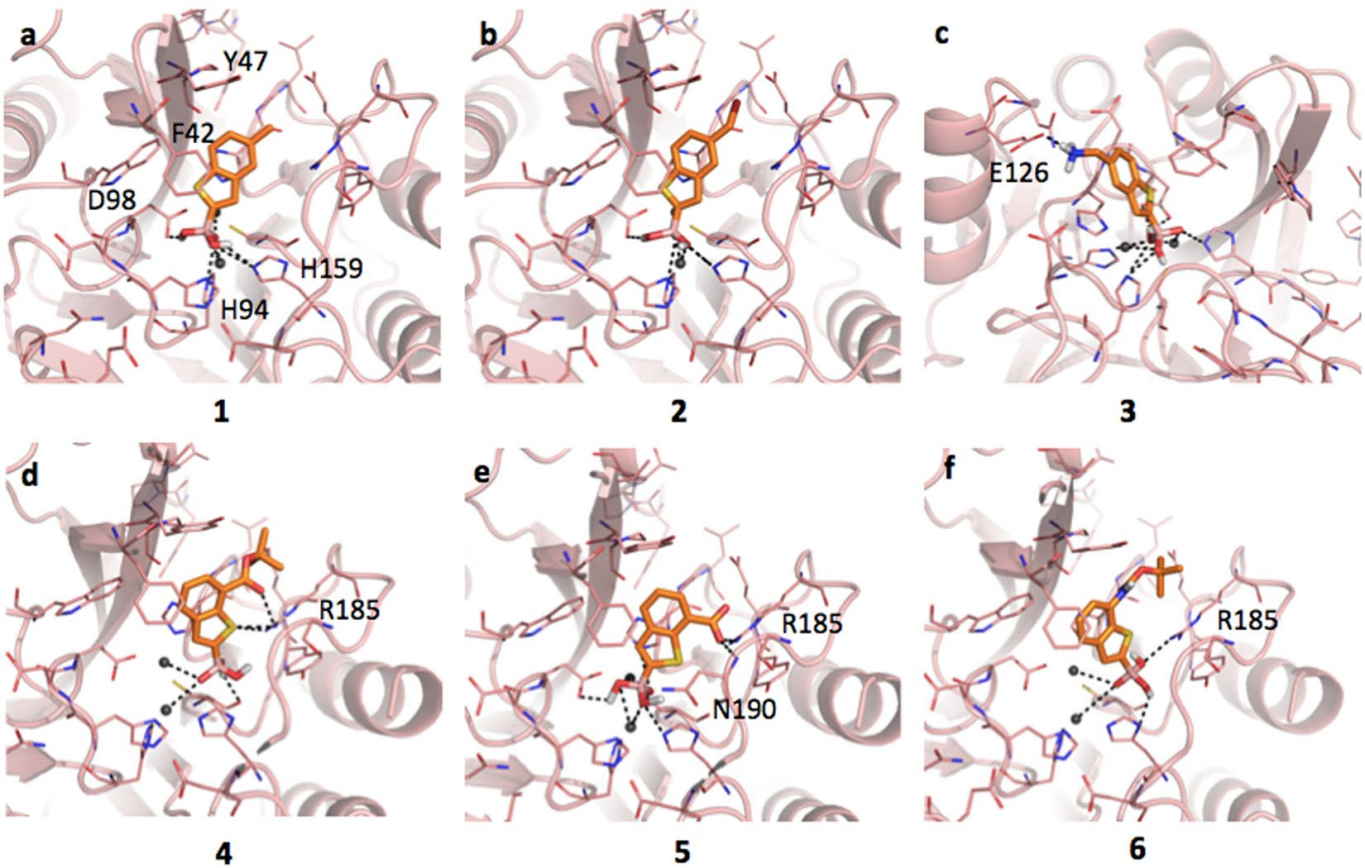
Docking poses for compounds in Table 1 in VIM-2 binding site (PDB code 2yz3). Residues lining the pocket are shown as capped sticks. Ligands are orange-coloured. Hydrogen bonds are represented by black dashed lines. Pictures were prepared using Pymol (http://www.pymol.org).

As mentioned, VIM-2 shows a binding site characterized by larger hydrophobic areas, mainly given by Phe42 and Tyr47, not present in NDM-1 (SI, Figure S1e,f). Compounds **1** and **2** present the same orientation and very similar IC_50_ values (72.00 μM and 83.50 μM, respectively). The boronic group coordinates the zinc ions through the deprotonated hydroxide and forms further interactions with His94, Asp98 and His159, also involved in the metals coordination. *Pi-pi* interactions are formed between the benzo(b)thiophene and the Phe42 side chain. The methyl and the methyl bromine moieties occupy the hydrophobic regions defined by the same residue (Fig. 7a and b, respectively). The ligand, and in particular the boronic group, present a quite similar orientation to that found by Brem *et al*. for cyclic boronates in VIM-2 (PDB code: 5FQC)^17^. Compound **3** presents an orientation within the pocket opposite to that of compound **1**, likely induced by the formation of a salt bridge between the protonated amine and Glu126. This additional interaction, besides the coordination and hydrogen bonds given by the boronic group, could explain the higher affinity of this compound (Fig. 7c; IC_50_ 26.90 μM). Similar inhibition values were also measured for compounds **4** and **5**, both able to hydrogen-bond Arg185 (Fig. 7d,e). In particular, compound **4** moves towards Arg185 forming a double contact through both the thiophen sulfur and the ester carbonyl. The metals coordination is maintained but the interactions with His94 and Asp98, as well as the *pi-pi* contact with Phe42, are lost. Compound **5** interacts with both the side chain of Arg185 and the backbone amide of Asn190 by means of the carboxylic moiety, still maintaining the *pi*-*pi* interaction with Phe42. Compound **6** assumes an orientation similar to compound **4** contacting Arg185 through the boronic group, keeping the metal coordination but not forming any other significant contact. This compound shows the weaker inhibition towards VIM-2 (Fig. 7f; IC_50_ 102.60 μM).

### Evaluation of the synergistic activity of selected compounds

In order to evaluate the usefulness of selected compounds for the design of phenotypic tests, and further support the results of the modeling and enzyme studies, we investigated the potential of boronic acids to inhibit bacterial β-lactamases in a whole-cell assay by acting in synergy with β-lactam antibiotics. As previously reported^22,46^, no antibiotic effect was detected for boronic acid alone (MIC higher than 128 μM). Compounds were tested for synergy in association with third-generation cephalosporins currently in therapy: ceftazidime (CAZ) against eight clinically isolated bacteria overproducing AmpC BL, and cefotaxime (CTX) against five clinically isolated bacteria strains hyperproducing CTX-M-15, KPC-2, NDM-1, VIM-2 and OXA-24 BLs (collection of the Spanish Network for Research in Infectious Pathology (REIPI, Spain), Table 2a,b). Selected strains naturally overexpressed the BLs in the panel, allowing the biological validation of the results obtained in the recombinant proteins assays. The minimum inhibitory concentrations (MICs), for CAZ and CTX alone and in combination with boronic acids against resistant strains were determined (Table 2a,b). Starting from isolates overproducing AmpC, the MIC of CAZ alone ranged from 512 μg/mL to 64 μg/mL (Table 2a,b)^51^. In combination with the BAs, the MIC values of CAZ decreased in several cases, by a minimum of 4 fold to a maximum of 32 fold for the best case.

**Table 2:**
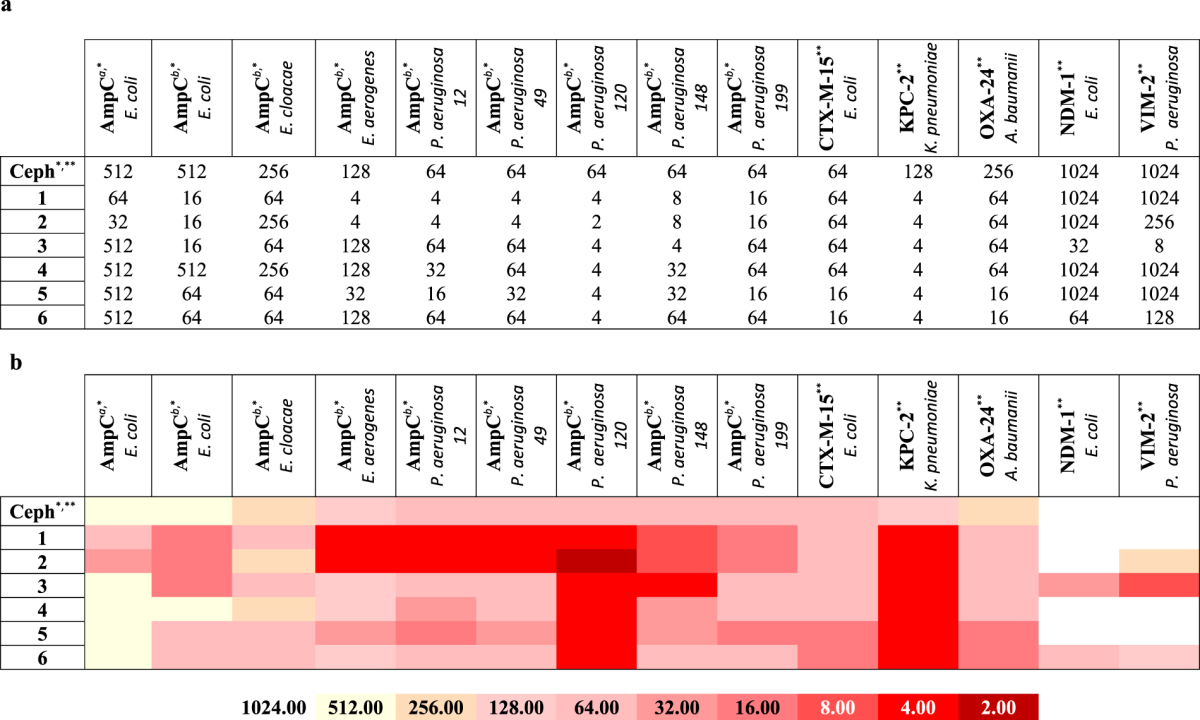
Boronic compounds panel antibacterial activity profile: a - Trending profile of antibacterial activity for the entire compounds library versus nine clinically isolated bacteria overproducing AmpC_BL (MIC values in combination with ceftazidime, CAZ,) and five clinically isolated bacteria strains producing class A (CTX-M-15, KPC-2), class B (NDM-1, VIM-2) and class D (OXA-24) BLs (MICs in combination with ceftazidime, CTX); b - Antibacterial activity heat-map: the Table shows, with a specific color scale, the MIC values trending profile for the entire compounds library as described above. ^a^Plasmidic AmpC - DHA1; ^b^Overproduction Chromosomal AmpC; *in combination with ceftazidime, CAZ; ** in combination with cefotaxime, CTX.

The best overall results were achieved with *E. coli, E. aerogenes* and *P. aeruginosa* strain 148. Analyzing each BA alone, for the combination CAZ/**1**, MICs improvement were always evident *vs* all considered clinical strains overproducing AmpC (from 4 fold up to 32 fold). **1** was able to restore the susceptibility of all strains overproducing *P. aeruginosa*. For the CAZ/**2** combination, **2** resulted slightly more active than **1** on the DHA-1-producing *E. coli* strain (16 fold *vs* 8 fold). Restoration of susceptibility against *P. aeruginosa* strains was evident as for compound **1**. On the contrary, no synergy was detected for the CAZ/**2** combination towards *E. cloacae*, while the association with **1** gave a 4-fold MIC decrease. Other derivatives (**3, 4-6**) showed, in general, poorer synergic effect with CAZ (16 fold, less or no MIC reduction). The only exception was compound **3**, reducing MIC by 32 fold on the AmpC hyperproducer *E. coli* strain and by 16 fold vs *P. aeruginosa* strain 148. The effect of the combination of the described BAs with CTX against six strains producing class A, B, C and D β-lactamases is more complex (Table 2a,b). The MIC of CTX for these pathogens ranged from 1024 to 64 μg/mL. Again, compounds **1** and **2** resulted the most active. Both compounds were able to improve CTX activity with almost all strains producing class A, B, C and D BLs, particularly in *P. aeruginosa* and *K. pneumoniae* (16 and 32 fold MIC reduction respectively). Only in the case of *E. coli* producing CTX-M-15 no synergic effect was detected, except for compounds **5** and **6 (**4 fold MIC reduction**)**. Also for compounds **3-6**, best synergistic effect was achieved *vs K. pneumoniae* producing KPC-2 (32 folds improvement). **3** showed the most potent synergistic activity, with a reduction of the MIC value up to 128-fold with all considered bacterial strains, except with CTX-M-15-producing *E. coli*. Overall, results for compounds **4**, **5** and **6** showed a null or very low effect, with the exception of compounds **5** and **6**, which gave the best performance towards *A. baumannii* strain producing OXA-24.

Interestingly, the synergistic activity of each selected BL inhibitor correlates to a certain extent with the inhibition potency measured with purified enzymes, also considering the difficulties in working with clinical strains, which commonly present additional resistance mechanisms, such as porins, efflux pumps etc. The distinctive activity profile of each compound in the panel could represent the basis for the design of a phenotypic test allowing the detection of specific types of clinically-relevant β-lactamases directly in clinical isolates.

## Discussion

**Figure 8:**
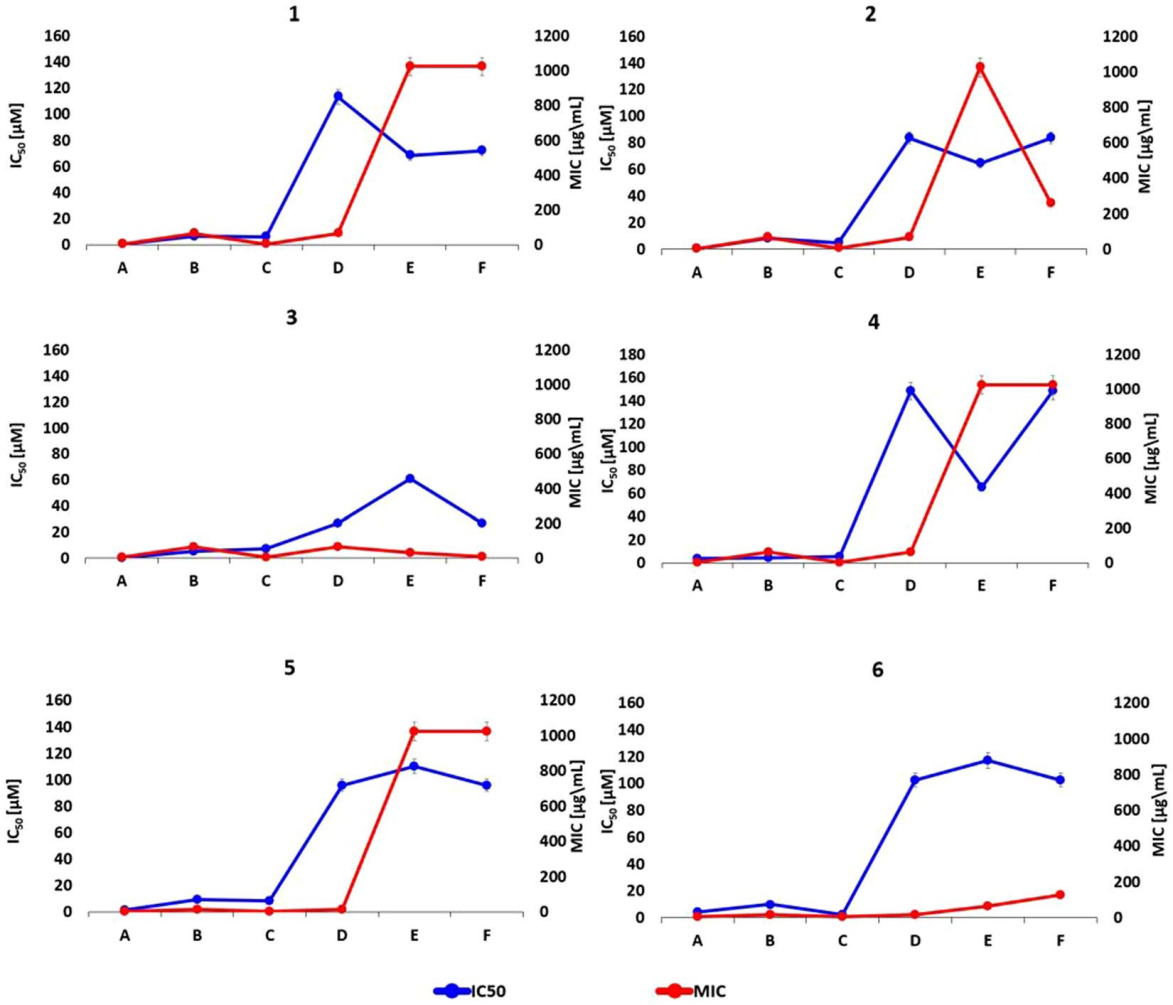
*In vitro* results for each designed compound against the selected BLs panel: IC_50_ and MIC values. Direct comparison between IC_50_ (*blue line*) and MIC (*red line*) values for each inhibitor of the selected compounds panel versus the several beta-lactamase proteins. The obtained data results show that each inhibitor presents a similar trending profile supporting a good correlation between *in vitro* datasets. The reported error bars correspond to 5% value for both datasets; ***A*** – AmpC Pseudomonas aeruginosa 120; ***B*** – CTX-M-15 E. coli; ***C*** – KPC-2 K. Pneumoniae; ***D*** – OXA-24 A. Baumanii; ***E*** – NDM-1 E. coli; ***F*** – VIM-2 Pseudomonas aeruginosa.

Six selected boronic acids were evaluated for their inhibitory activity against clinically-relevant serine- and metallo-β-lactamases, as well as their potential usefulness for the design of detection tool to be used in the clinical microbiology laboratory. To this aim, the antibacterial synergistic activity of these inhibitors was measured with a set of clinical isolates producing various β-lactamases, and the data compared to the enzyme inhibition potencies. In this respect, the boronic acids derivatives were first evaluated with enzyme assays against a panel of relevant BLs, then *in silico*, and finally with antibacterial susceptibility testing methods. From a computational point of view, the challenge of a feasible characterization of the covalent binding resulting between the boronic acid moiety and the catalytic serine has been addressed^52,53^. The formation of a reversible covalent bond generally increases the binding affinity to an extent lower than 20 fold. Therefore it does not always lead to a tighter binding and, most important, an equal contribution to the protein-ligand complex formation and stabilization could be given by other geometrical and non-covalent energetic factors^54^. In this perspective, for the analyzed series of boronic acids the effect of substituents could be almost as important as the effect of the boronic group itself as seen from the different binding affinity shown towards selected BLs. Compounds **1**, **2** and **3** show high nanomolar inhibitory potency towards AmpC (IC_50_ 0.4 μM; 0.2 μM and 0.6 μM respectively, Table 1a,b). This result accounts for the higher efficacy of derivatization in position C5 with respect to C7 *vs* AmpC. As the covalent binding mechanism does not change and all AmpC-BA acyl enzymes maintain the same deacylating orientation, geometrical and non-covalent factors should explain variation in affinity^21,46^. The analysis of the docking results reveals in AmpC the possibility of assuming two slightly different orientations, one favoring the formation of a *pi-pi* contact with Tyr221 and the other forming a dipole-quadrupole interactions with Asn152. This might justify the higher affinity because of a smaller entropic loss upon binding^47^. Accordingly, the presence of an aromatic system has been proven to be more critical in targeting class C BLs, with respect to class A. Compounds **4**, **5** and **6** loose this flexibility because of the presence of bulkier substituents in C7, which force ligands to move apart from Tyr221. The different inhibition trend and the positive effect of C5 derivatization type are clearly visible in Table 1a,b. In particular each compound shows a peculiar inhibition profile against the BLs in panel supporting their possible future use in combination as diagnostic tools (Table 2a,b). Compounds binding to class A CTX-M-15 and KPC-2 show a similar trend (Fig. 8). Here the covalent bonding generates an acyl-enzyme, further stabilized by a number of H-bonds between the boronic group and the surrounding residues. The loss in affinity with respect to AmpC, considering in particular compounds **1**, **2** and **3** (IC_50_ vs KPC-2 5.9 μM; 4.2 μM and 7.3 μM respectively), can be attributed to three interconnected aspects: (i) absence of a strong *pi-pi* contact, (ii) absence of a quadrupole-dipole interaction, (iii) reduced compound flexibility in the binding site (Table 1a,b).

For the biological evaluation of the selected boronic acids, a set of representative clinical isolates of *E. coli*, *E. cloacae*, *E*. *aerogenes*, *P. aeruginosa*, *K*. *pneumoniae* and *A. baumanii*, showing low susceptibility to CAZ and CTX, and producing the β-lactamases included in the study was obtained^55^. *In vitro* antimicrobial susceptibility testing showed a valuable synergistic activity of the selected compounds, although the antibiotic potentiation activity varied in function of the compound and the clinical isolate. Interestingly, a good correlation was observed between the synergistic activity and the enzyme inhibition profile of the various compounds. We compared the biological profile of each boronic acid versus BLs as IC_50_ values (Fig. 8, blue line) and bacteria as MIC values (Fig. 8, red line). Regarding to the several overproducing AmpC_BL bacterial strains, we selected *Pseudomonas aeruginosa 1*2*0* as the most representative of the compounds panel efficacy in inhibiting bacterial growth. We consider that MIC higher than 128 μM represents low inhibitory effect on the cell growth (8 times reduction of CTX IC_50_). IC_50_ higher than 30 μM indicates low inhibitory effect against BL enzyme. We can observe that for each boronic acid under study there is a similar trend for compounds **1**/**5** and **2**/**4** considering almost all proteins (CTX-M15, KPC-2, NMD-1 and OXA-24). They show however different potency. Compounds **3** and **6** show also a similar trend, but with different potency.

The distinctive inhibition profile and synergistic activity of the selected compounds could represent the basis for the development of a detection tool able to identify major β-lactamases, including carbapenemases, currently circulating in the clinical setting, although further validation is required. Nonetheless, we were able to correlate chemical and binding features of the inhibitors with their inhibition profile. Noteworthy, for the very first time, acyclic boronates derivatives were reported to inhibit MBLs via a non covalent mechanism of action. These results, considering their described ability to inactivate all classes of serine-β-lactamases, open the way for developing BAs as very broad spectrum inhibitors.

Summarising, the C5 derivatization favors AmpC inhibition, with compounds **1** and **2** being able to potentiate the antibiotic activity towards several AmpC producing strains (Table 2; Fig. 8). Substituents in C7 position allowed better synergistic activity towards CTX-M-15-producing strains, while C5 derivatives gave better results towards OXA-24-producing *A. baumannii*. (Table 2; Fig. 8).

An additional relevant result of our study is represented by the ability of some compounds to potentiate the activity of cefotaxime on carbapenemase-producing *P. aeruginosa* or *A. baumannii* isolates, usually poorly inhibited by currently available combinations, including ceftazidime-avibactam, despite their rather high clinical relevance^56,57^.

## Conclusions

Boronic acids represent a known class of investigational and therapeutically valuable BLIs potentiating the activity of β-lactam antibiotics. Besides their therapeutic applications, the development of specific detection tools represents an additional field of application of boronic acids. From an integrated approach aiming at evaluating the interaction of a boronic acids set against a selected BLs panel including clinically-relevant AmpCs, ESBLs, serine and metallo-carbapenemases, we identified C5-substituted BZB derivatives. Covalent docking studies, enzyme inhibition analysis and biological evaluations were performed. The synthesized boronic acids showed high nanomolar affinity towards class C AmpC BL and low micromolar affinity towards class A CTX-M-15 and KPC-2 enzymes. Interestingly for the first time inhibition activity against MBLs (NDM-1 and VIM-2; IC_50_ range 20 µM in the best case) was also detected for acyclic boronic acids: noteworthy the activity of cefotaxime on a VIM-2 producing *P. aeruginosa* isolate in microbiological assays was potentiated by 32 fold, despite their inhibition potency *in vitro* (IC50 values in the µM range). Docking studies supported the observed structure-activity relationships, confirming the predictive capability of the computational study. Indeed, a newly ad hoc covalent docking algorithm was implemented within FLAPdock, representing a valuable tool for investigating the inhibitory activity of covalently-bound inhibitors, being able to identify the chemical features responsible for the specific inhibition profile of the compounds towards various β-lactamases. Antimicrobial susceptibility testing confirmed the capability of selected compounds to act in synergy with third-generation cephalosporins against multidrug-resistant clinical isolates, with observed potentiation fold up to 256. The enzyme inhibition profile satisfactorily correlated with the synergistic activity measured in whole-cell assays.

The data presented confirmed the possibility to discriminate between clinically-relevant β-lactamases on the basis of the inhibition profile of each compound belonging to the designed multiligand set. Our results open the way to the potential use of our designed compounds set as a diagnostic tool for sensitive detection of clinically-relevant β-lactamases.

## Material and Methods

### Protein expression and purification

The *P. aeruginosa* AmpC β-lactamase was obtained from *E. coli* BL21(DE3) culture carrying the plasmid vector pET-9aAmpC in 1 L of LB-medium with kanamycin 50 μg/mL, at 37 °C, 150 RPM for 24 hrs. 3-methylaminophenylboronic acid (MAPB) was used to functionalize Affigel-10 resin (BIORAD) as previously described^58,59^. In order to produce and purify the other β-lactamase enzymes, the β-lactamases ORFs were subcloned in various expression vectors such as pET-9a (for CTX-M-15, KPC-2 and VIM-2), pET-15b (for NDM-1) or pGEX-2T (for OXA-24) and introduced in Escherichia coli BL21(DE3) or DH5α. Culture of ZYP-5052 auto-inducing medium or SB were grown for 24 hours at 25 or 37 °C under selective condition (50 µg/ml kanamycin or 100 µg/ml ampicillin according to the vector carried by the recombinant strain). Cells were harvested by centrifugation (10,000 × g, 30 min, 4 °C). KPC-2, NDM-1 and OXA-24 were purified from the crude extract while CTX-M-15 and VIM-2, from the culture supernatant. The β-lactamases were purified to >95% homogeneity by ion-exchange, gel filtration or affinity chromatography adopting specific purification strategies using an AKTA Purifier platform (GE Healthcare, Uppsala, Sweden). Purity of the protein preparation was estimated by SDS-PAGE analysis and electron spray ionization mass spectrometry (ESI-MS)^60^. For each protein, kinetic profile characterization was conducted using different β-lactam antibiotics depending on belonging BL class; *i.e*. cephalothin for class A CTX-M-15, class C AmpC and class D OXA-24, ampicillin for class A KPC-2, imipenem for class B NDM-1 and VIM-2. A detailed description of protein purification protocols and protein kinetic profile characterization is given in the Supplementary Information. (SI, Table S1).

### Enzyme inhibition assays

The synthesized compounds were tested against the panel of purified β-lactamases, *i.e*. AmpC, CTX-M-15, KPC-2, OXA-24, NDM-1 and VIM-2 by spectrophotometric assay, using a DU-640 spectrophotometer (Beckman Coulter)^20^. Boronic acids were dissolved in dimethyl sulfoxide (DMSO) stock solutions at 10 mM; more dilute stocks were subsequently prepared as necessary by dissolving them in 50 mM phosphate buffer at pH 7.5 (DMSO ≤ 3% V/V, no inhibitory effect confirmed). Boronic acids isolated as pinacol-protected boronic acid were tested without further ester cleavage reaction; compounds spontaneously hydrolysed to the free acid form when dissolved in 50 mM phosphate buffer at pH 7.5. Assay conditions were as follows: 50 mM phosphate buffer, pH 7.5; 100 *μ*M cephalothin (sodium salt, Sigma) as reporter substrate, reaction monitored at 265 nm, time course 300 seconds 25 °C. The background rate of cephalothin hydrolysis was found to be negligible under these conditions (approximately 1%). Reactions were initiated with addition of BL enzyme (the concentration values for each BL enzyme into the SI, Table S1 have been reported).

Each inhibitor compound was assayed at five different concentrations, in *duplicate* for calculating an error value with 95% confidence interval (ρ < 0.05). From the resulting inhibition percentages at each different inhibitor concentration and, assuming a competitive inhibition for the boronic compounds panel, it was possible to calculate the IC_50_ values from the Dixon plot 1/V vs [I], using the equation IC_50_ = ((1/0.5 ∙ v_0_) − m)/q^61^, where v_0_ is the rate of hydrolysis of the reporter substrate (v_0_ being the rate measured in the absence of inhibitor), *q* the y axis intercept and *m* the slope of the resulting linear regression. As previously reported for other boronic acid inhibitors of BLs, tested compounds were competitive inhibitors and no incubation effect was detected^21,22^. A known inhibitor was included in the compounds panel for each β-lactamase group, *i.e*. 3APBA (3-aminophenylboronic-acid) for class *A*, *C* and *D* serine β-lactamases (IC_50_ = 25 ± 1.9 µM for AmpC_BL^44^) and L-Captopril for class *B* β-lactamases as reference compound (IC_50_ = 154 ± 1.7 µM for NDM-1)^62^.

### Molecular docking

FLAPdock is a docking approach implemented in the software FLAP^35^, based on GRID Molecular Interaction Field similarities^36,63^, combined with classical energetics. FLAP has been applied in different drug design campaigns, giving successful performances in terms of structure-, ligand- and pharmacophore-based virtual screening^64–69^. The FLAPdock method and full validation is as yet unpublished; hence a detailed description of the method is provided below. FLAPdock follows a molecular fragmentation approach, subsequent placement of each fragment in the site of the target, followed by incremental construction of the molecule. Ligand fragments are generated using an iterative procedure searching for substructures with 7–12 heavy atoms and 1–3 rotatable bonds; these parameters having been chosen to provide fragments that are small enough to reduce the conformational space and increase the speed of the placement algorithm, and large enough to contain a certain level of specificity in the site. If no fragments are found, these rules are relaxed, and subsequently, if no fragments are found again, then the ligand is treated as a single entity. Fragment conformations are taken from the previous ligand conformational ensemble and a further RMS clustering performed to remove any redundancy. This approach is performed (as opposed to performing a conformational search only on the fragments) to focus fragment conformations toward those that are able to present once the entire ligand is reconstructed. Each fragment is used as a starting point for the docking, unless it is completely un-functionalized (i.e. it must contain a donor/acceptor/charged group). A set of poses for the starting fragment is generated using FLAP quadruplet alignment of the fragment conformer atom quadruplets, and the receptor site GRID MIF minima points; in this way hundreds of thousands of poses are typically generated for this starting fragment. As a first step, the poses are scored using a weighted sum of the FLAP field similarities, including shape, donor, acceptor, and hydrophobic similarity. This faster scoring enables the selection of a subset of poses that are more likely to contain the ‘correct’ position for this fragment; typically several hundred solutions are retained per conformation, and therefore several thousand over the fragment ensemble. A second scoring step calculates Lennard-Jones and dielectric corrected Coulombic energetic terms for this subset; the solutions are then ranked according to the combined score and, subjected to RMS clustering, and the best scoring pose in each cluster retained. The best 100–200 solutions from this first step (after docking all fragments) are typically retained for the subsequent phase (depending on user request). For each solution, the subsequent fragment is added at 12–18 random torsional angles. In this phase, added fragments can be quickly filtered according to any internal or external clashes, before scoring according to the field similarity and energetic terms. The final pool of solutions is subjected to RMS clustering and filtering, followed by retaining of the top 100–200 solutions. The final phase occurs when the ligand is completely reconstructed; at this point the top 10 solutions are subjected to energetic optimization in the site, and rescored after recalculating the ligand MIFs for this conformation. Internal validation (redocking of x-ray ligands) has shown that one of the top five poses contains a pose within 2.0 Å of the x-ray position in more than 90% of the cases [unpublished results]. For the work described in this paper, a modification of the approach was developed to simulate the covalent interaction between the β-lactamase catalytic serine and the boronic acid. The simulation is not trivial, due to the conformational change of the boronic acid from an *sp* ^*2*^ to *sp* ^3^ configuration. To do this, the library of boronic acids was imported into a virtual reaction software, and the reaction with a serine residue simulated to generate a library of boronic acyl-serine compounds. Within FLAPdock, each compound in the library was docked, where the boronic acyl-serine was subjected to the conformational preparation procedure outlined above; in this way the correct geometry around the boronic acid was maintained. During the fragmentation step, the serine substructure was used to generate the initial fragment (and it’s position, without scoring), and then the subsequent fragments from the boronic acyl-serine structure added incrementally as described above.

In the case of NDM-1 and VIM-2 the compounds were directly docked in the *sp* ^3^ configuration, following the indication provided by Brem *et al*.^17^. The standard FLAPdock approach was used since no covalent interaction is formed.

### Biological evaluation

Susceptibility testing was performed in Mueller Hinton broth and interpreted following the guidelines of the National Committee for Clinical Laboratory Standards^70^. To test the inhibitory activity, the compounds were dissolved in DMSO, 50% in water, and further dilutions were performed using growth medium. In all cases tests were performed at a final DMSO concentration below 5%, avoiding unwanted DMSO inhibition effect on strains growth (blank test control with DMSO were performed). The MICs of cefotaxime and ceftazidime alone and in association were determined against several relevant Gram-negative clinical isolates, selected from the pathogen collection of the Spanish Network for Research in Infectious Pathology (REIPI; Spain). The selection was based on strains capability of producing β-lactamases of interest for the present study. The antibiotic:inhibitor ratio was 1:1 (w/w).

### Data availability

The datasets generated during and/or analysed during the current study are available from the corresponding author on reasonable request.

## Electronic supplementary material

Supplementary Information
