# Supplementary material for: Computational and biological profile of boronic acids for the detection of bacterial serine- and metallo-β-lactamases
Source: Sci Rep. 2017 Dec 18;7:17716. doi: 10.1038/s41598-017-17399-7 (PMC5735191; doi:10.1038/s41598-017-17399-7)
Supplement: Supplementary file 1 — Supplementary Information [file 41598_2017_17399_MOESM1_ESM.pdf]

Computational and biological profile of boronic acids for the detection of bacterial serine- and metallo-β-lactamases

Matteo Santucci[1,#], Francesca Spyrakis[1,2,#], Simon Cross[3#], Antonio Quotadamo[1], Davide Farina[1], Donatella Tondi[1], Filomena De Luca[5], Jean-Denis Docquier[5], Ana Isabel Prieto[6], Claudia Ibacache[7], Jesús Blázquez [6,7], Alberto Venturelli[1,4,]*, Gabriele Cruciani[8,]*, Maria Paola Costi[1,]*

[1] Department of Life Sciences, University of Modena and Reggio Emilia, Via Campi 103, 41125, Modena, Italy; [2] Current Address: Department of Drug Science and Technologies, University of Turin, Via Pietro Giuria 9, 10125, Turin, Italy; [3] Molecular Discovery Limited, 215 Marsh Road, Pinner Middlesex-London HA5-5NE, United Kingdom; [4] TYDOCK PHARMA S.r.l., Strada Gherbella 294/b, Modena, 41126, Italy; [5] Dipartimento di Biotecnologie Mediche, University of Siena, Viale Bracci 16, 53100, Siena, Italy; [6] Biomedicine Institute of Sevilla (IBIS)-CSIC, Avda. Manuel Siurot, sn. Sevilla, Spain; [7] [7]National Center of Biotechnology-CSIC, Calle Darwin, 3, 28049-Madrid, Spain; [8] Department of Chemistry, Biology and Biotechnology, University of Perugia, Via Elce di Sotto 8, 06123, Perugia, Italy.

Supplementary Material

Table of Content

**Protein expression and purification**	**Pag. 3**
**Table S1. Protein purification methods and characterization details**	**Page 6**
**Figure S1. Overall folding and binding site Molecular Interaction Fields for AmpC *(a)*, CTX-M-15 *(b)*, KPC-2 *(c)*, OXA-24 *(d),* NDM-1 *(e)* and VIM-2 *(f)***	**Pag. 7**
**Table S2. NMR characterization and atoms numbering of the synthesized compounds**	**Pag. 8**
**Scheme S1. Synthesis**	**Pag. 10**
**Chemical synthesis**	**Pag. 11**
**Scheme S2. Mechanism of action**	**Pag. 16**
**Figure S2. Possible orientation assumed by compound 2 in AmpC binding site**	**Pag. 17**
**References**	**Pag. 18**

Protein expression and purification

AmpC. *P. aeruginosa* AmpC-β-lactamase was obtained from a culture of *E. coli* BL21(DE3), carrying the plasmid vector pET-9aAmpC in 1L of LB-medium with kanamycin 50 μg/mL, at 37 °C, 150 RPM for 24 hrs. The OD_{600nm} was checked every hour, at a 0.6-0.8 value protein expression was induced adding *3-D-isogalactopyranoside* (IPTG) (1 mM) and incubating the culture for 6 h, at 37°C, 150 RPM. After 6 hours the cell broth was centrifuged at 4500 RPM, 4 °C, for 20 min. The supernatant was recovered, concentrated by ultrafiltration (Centricon Plus-70, Millipore), and desalted using a Hi-Prep desalting 26/10 column (GE Healthcare) with load-buffer (0.02 M Tris; 0.5 M NaCl; pH 7.0). The sample was then loaded on a XK 16/20 column packed with 25 ml of Affigel-10 (Biorad) functionalized with 3 MAPB (3-methylaminophenylboronic acid). The column was previously equilibrated with the load-buffer. The protein was eluted (flow rate, 1 mL/min) with 5 column-volumes of elution-buffer (0.5 M boric acid, 0.5 M NaCl, pH 7.0). β-lactamase activity was checked in each elution fraction using 100 μM cephalothin. Active fractions were pooled, concentrated by ultrafiltration using a Millipore Ultra-15 (Ultracel-10K; Millipore) and checked by SDS-Page. The protein pool final concentration (1mg/mL) was assessed using Bradford and UV-Vis assays. Purified AmpC β-lactamase (1 mg/mL) was stored at -80 °C.

CTX-M-15. CTX-M-15 β-lactamase was obtained from a culture of *E. coli* BL21(DE3). The BL21(DE3)/pET-CTX-M-15 strain was grown aerobically in ZYP-5052 medium, in presence of 50 μg/mL kanamycin at 37°C for 24 hrs[1]. The culture was centrifuged at 4,500 rpm, 4 °C, for 20 min and the culture supernatant was concentrated using ultrafiltration (Amicon model 2000, Millipore membrane YM10). The resulting sample was desalted (HiPrep 26/10 Desalting column, GE Healthcare) using 10 mM HEPES (pH 7.4) (buffer A). The resulting sample was loaded onto a 5 mL HiTrap SP Fast Flow column (flow rate, 2 mL/min) previously equilibrated with Buffer A. The protein was eluted using linear NaCl gradient in Buffer A (0 to 150 mM NaCl in 100 mL). The active fractions were pooled and stored at -20 °C until use. β-lactamase activity was monitored by determining the initial rate of hydrolysis of 100 μM cephalothin. The authenticity and purity of the final protein sample was confirmed by ESI-MS and SDS-PAGE.

KPC-2. Strain BL21-DE3/pET9-KPC2 was grown in 2 L of ZYP-5052 auto-inducing medium for 24 h at 37 °C. The culture was centrifuged at 4,500 rpm, 4 °C, for 20 min. The bacterial cells was resuspended in 200 mL of buffer A (20 mM Tris-HCl, pH 8.5) and lyzed by sonication (Sonopuls HD-2200). The cell extract was clarified by centrifugation (12,000 rpm, 4 °C, 30 min), desalted (HiPrep 26/10 Desalting column) with buffer A, and loaded on a 25-ml Q-Sepharose Fast Flow

column (flow rate, 2 mL/min) previously equilibrated with the same buffer. The protein was eluted using a NaCl gradient in buffer A (0 to 100 mM NaCl in 500 mL). The active fractions were pooled, concentrated using Amicon Ultra 15 (MW cut off, 10 kDa) and desalted as before using buffer B (50 mM MES [morpholineethanesulfonic-acid], pH 6.0). The desalted sample was loaded onto a 5mL HiTrap SP High Performance column (flow rate, 2 mL/min), previously equilibrated with buffer B. The β-lactamase was eluted in the flow-through, concentrated by ultrafiltration, and loaded onto a MonoQ 5/50 GL column (flow rate, 2 mL/min), previously equilibrated with buffer C (10 mM HEPES, pH 7.5). The protein was eluted using a linear NaCl gradient in buffer C (0 to 140 mM NaCl in 50 mL). β-lactamase activity was monitored using the hydrolysis of 1 mM ampicillin. The authenticity and purity of the final protein sample was confirmed by ESI-MS and SDS-PAGE.

OXA-24. OXA-24 β-lactamase was produced in *E. coli* as a N-terminal GST fusion protein using a pGEX-2T plasmid vector. Strain *E. coli* DH5α/pGEX-2T-OXA-24 was grown in 1 L of Super Broth medium for 24 h at 37 °C[2]. Cells were recovered by centrifugation and resuspended in 100 mL of 50 mM Tris-H_2SO_4 supplemented with 0.1 M K_2SO_4 buffer (pH 7.5) (buffer A). Cells were lyzed by sonication and the extract clarified by centrifugation at 12,000 rpm, 4 °C, for 20 min. The sample was loaded on a pre-packed 5-mL GSTrap Fast Flow column at a flow rate of 0.5 mL/min, previously equilibrated with buffer A. The protein was eluted using in the presence of 10 mM reduced glutathione in buffer A. The active fractions were dialyzed against buffer A. The N-terminal GST was cleaved in the presence of thrombin (1 U/μL). The resulting sample was loaded on a HiTrap Benzamidine Fast Flow column (1 mL) at a flow rate of 0.2 mL/min. β-lactamase activity was monitored using the hydrolysis rate of 100 μM cephalothin. The authenticity and purity of the final protein sample was confirmed by ESI-MS and SDS-PAGE.

VIM-2. The VIM-2 metallo-β-lactamase was purified from a 2 L culture of BL21-DE3/pET9-VIM-2 as previously described[3]. Briefly, the protein was purified from the culture supernatant, concentrated by ultrafiltration, and purified using two anion exchange chromatography steps, followed by a final gel filtration step, as described. β-lactamase activity was monitored using the hydrolysis rate of 150 μM imipenem. The authenticity and purity of the final protein sample was confirmed by ESI-MS and SDS-PAGE.

NDM-1. *E. coli* strain BL21(DE3)/pET-15-NDM-1 was grown in 1 L of ZYP-5052 medium for 24h at 37 °C. Cells were recovered by centrifugation (4,500 rpm, 4 °C, 20 min) and resuspended in 200 mL of buffer A (10 mM Hepes, 0.15 M NaCl, pH7.5). The cells were lyzed by sonication

(Sonopuls HD-2200) and the cell debris removed by centrifugation (12,000 rpm, 4 °C, 30 min) in the presence of Protease Inhibitor Complete (Roche). The clarified sample was loaded onto a 5mL HisTrap Fast Flow column (GE Healthcare). The protein was eluted with imidazole (350 mM in buffer A) The active fractions were pooled, concentrated by ultrafiltration (Amicon Ultra 15, MW cut-off, 10 kDa), desalted (Hi Prep 26/10 Desalting) in buffer B (20mM triethanolamine, 50 μM $ZnSO4$, pH 7.2) to remove imidazole. β-lactamase activity was monitored using the hydrolysis rate of 150 μM imipenem. The authenticity and purity of the final protein sample was confirmed by ESI-MS and SDS-PAGE.

Table S1.

β -lactamase	Purification Method	β -lactam substrate	Km [µM]	kcat [s^{-1}]	E [µM]	Kcat/Km ($\mu M^{-1} s^{-1}$)
AmpC	***One step*** [4]<br>3APBA Affinity	Cephalothin	33 ± 2	159 ± 32	0.019	4.82
CTX-M-15	***Two steps*** [5]<br>Anion Exchange: Q-Sepharose<br>Gel Filtration: Sephadex G75	Cephalothin	41 ± 3	35 ± 7	0.067	0.85
KPC-2	***Three steps*** [6]<br>Anion Exchange: Q-Sepharose<br>Cation Exchange: SP-HP 16/10<br>Cation Exchange: MONO-Q 5/50	Ampicillin	55 ± 7	200 ± 40	0.047	3.64
OXA-24	***Two steps*** [7]<br>GST Affinity; HiTrap Benzamidine FF Affinity	Cephalothin	20 ± 2	1000 ± 150	0.035	50.00
VIM-2	***Three steps*** [8]<br>Anion Exchange: Q-Sepharose<br>Anion Exchange: Resource Q<br>Gel Filtration: Sephadex G75	Imipenem	11 ± 3	8 ± 2	0.043	0.72
NDM-1	***One step*** [9]<br>His-Tag Affinity	Imipenem	58 ± 6	520 ± 84	0.021	8.97

Protein purification methods and kinetic profile characterization details: the Table reports the *step-by-step* purification procedure for each β-lactamase protein, indicating the β-lactam substrate used for kinetic profile characterization (*kcat*, *Km* and *specificity constant* evaluation) and the proper concentration of enzyme used in the assay for each BL class.

Figure S1

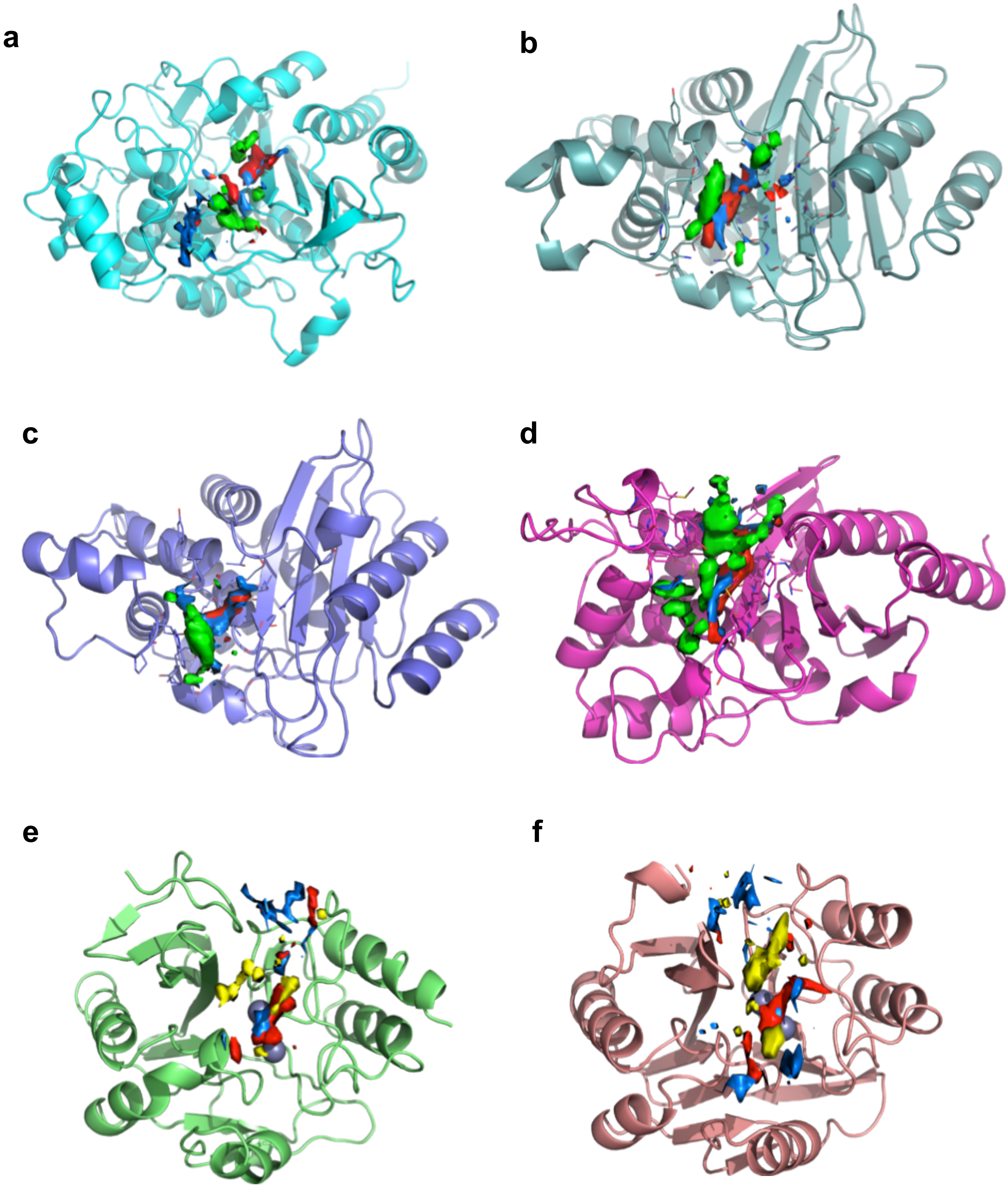

Overall folding and binding site Molecular Interaction Fields for AmpC *(a)*, CTX-M-15 *(b)*, KPC-2 *(c)*, OXA-24 *(d),* NDM-1 *(e)* and VIM-2 *(f)*. PDB codes 1kdw, 4hbt, 3rxx, 3fv7, 3q6x, 2yz3, respectively. The green, blue and red contours respectively identify the hydrophobic, H-bond donor and H-bond acceptor Molecular Interaction Fields (MIFs) as calculated by GRID. The grey spheres in e and f identify the active site zinc ions.

Table S2. NMR characterization and atoms numbering of the synthesized compounds.

Compound number	Structure	^{1}H-NMR	^{13}C-NMR
1	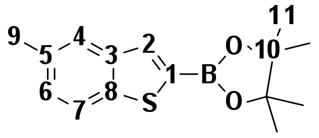	(DMSO): *δ* 1.42 (s, 12H, H-11), 2.53 (s, 3H, H-9), 7.25(d, *J=8.2 Hz*, 1H, H-6) 7.68 (s, 1H, H-4), 7.82 (d, *J=8.2 Hz*, 1H, H-7), 7.84 (s, 1H, H-2).	(DMSO): δ 21.4 (C-9), 24.8 (C-11), 84.4 (C-10), 122.1 (C-7), 124.1 (C-4), 127.3 (C-6), 133.8 (C-2), 134.1 (C-5), 140.9 (C-8), 141.0 (C-3). (quaternary CB not seen).
2	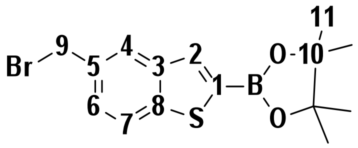	(DMSO): δ 1.39 (s, 12H, H-11), 4.65 (d, 2H, H-9), 7.42 (dd, 1H, H-6), 7.88 (m, 3H, H-2, H-4, H-7).	(DMSO): 24.7 (C-11), δ 30.8 (C-9), 85.4 (C-10), 121.9 (C-7), 124.7 (C-4), 126.9 (C-2), 137.2 (C-6), 137.6 (C-5), 140.8 (C-8), 141.1 (C-3). (quaternary CB not seen).
2a	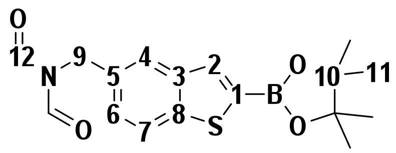	(DMSO): δ 1.38 (s, 12H, H-11), 4.91 (s, 2H, H-9), 7.41 (dd, 1H, H-6), 7.88 (m, 3H, H-2, H-4, H-7), 8.91 (br s, 2H, H-12).	($CDCl_3$): δ 21.9 (C-9), 25.7 (C-11), 85.0 (C-10), 122.8(C-7), 124.8 (C-6), 128 (C-2), 134.4 (C-4), 134.8 (C-3), 141.6 (C-8), 141.8 (C-5). C-1 and C-12 were not seen.
3	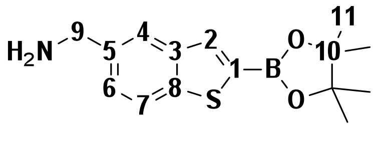	(DMSO): δ 1.31 (s, 12H, H-11), 4.15 (s, 2H, H-9), 7.55 (dd, 1H, H-6), 7.92 (s, 1H, H-4), 8.03 (s, 1H, H-2), 8.08 (d, 1H, H-7), 8.38 (br s, 2H).	(DMSO): δ 24.4 (C-11), 42.1 (C-9), 84.3 (C-10), 122.7 (C-7), 124.9 (C-6), 126.4(C-2), 130.2 (C-4), 134.2 (C-3), 139.9 (C-8), 142.6 (C-5), (quaternary *C*B not seen).
4a	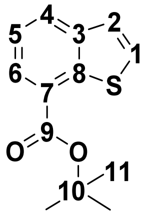	(DMSO): *δ* 1.73 (s, 9H, H-11), 7.43 (d, 1H, *J=5.5 Hz*, H-2) 7.48 (t, 1H, *J=7.6 Hz*, H-5), 7.60 (d, 1H, *J=5.6 Hz,* H-1), 8.03 (dd, *J=7.8 Hz, 1.3 Hz*, 1H, H-4), 8.13 (dd,	(DMSO): δ 28.3 (C-11), 82.1 (C-10), 122.7 (C-1), 123.5 (C-5), 126.0 (C-7), 126.8 (C-6), 127.7 (C-4), 128.7 (C-2), 139.5 (C-8), 140.9 (C-3) 165.3 (C-9).

		J=7.5 Hz, *1.3 Hz*, 1H, H-6).	
**4**	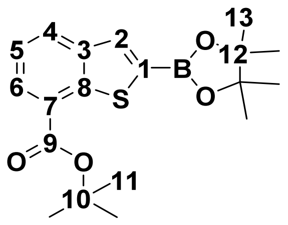	(DMSO): *δ* 1.41 (s, 12H, H-13), 1.72 (s, 9H, H-11), 7.46 (t, 1H, *J=7.6 Hz*, H-5), 7.95 (s, 1H, H-2), 8.05 (dd, *J= 7.9 Hz, 1.3 Hz*, 1H, H-4), 8.14 (dd, *J=7.4 Hz, 1.1 Hz*, 1H, H-6).	(DMSO): δ 24.8 (C-13), 28.3 (C-11), 82.1 (C-10), 84.5 (C-12), 123.6 (C-5), 126.2 (C-7), 127.7 (C-6), 128.6 (C-4), 133.4 (C-2), 141.6 (C-3), 143.5 (C-8), 165.2 (C-9). (quaternary *C*B not seen).
**5**	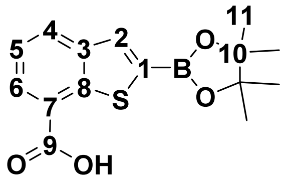	($CDCl_3$): *δ* 1.34 (s, 12H, H-11), 7.55 (t, 1H, *J=7.6 Hz,* H-5), 7.98 (s, 1H, H-2), 8.10 (dd, *J= 7.4 Hz, 1.1 Hz*, 1H, H-4), 8.21 (dd, *J=7.8 Hz, 1.1 Hz*, 1H, H-6).	($CDCl_3$): δ 21.1 (C-11), 122.1 (C-7), 123.3 (C-1), 123.4 (C-4), 125.9 (C-6), 126.2 (C-2), 133.8 (C-5), 137.0 (C-8), 139.9 (C-3). C-1 and C-9 were not seen.
**6**	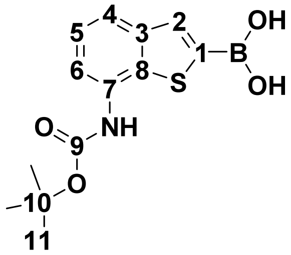	(DMSO): *δ* 1.56 (s, 9H, H-11), 7.34 (t, 1H, *J=7.7 Hz*, H-5), 7.46 (d, *J= 7.5 Hz*, 1H, H-6), 7.68 (d, *J=7.8 Hz*, 1H, H-4), 7.88 (s, 1H, H-2).	(DMSO): δ 27.4 (C-11), 79.5 (C-10), 119.1 (C-6), 120.6 (C-4), 124.3 (C-5), 132.5 (C-7), 133.0 (C-2), 137.2 (C-8), 141.9 (C-3). C-1 and C-9 were not seen.

Scheme S1.

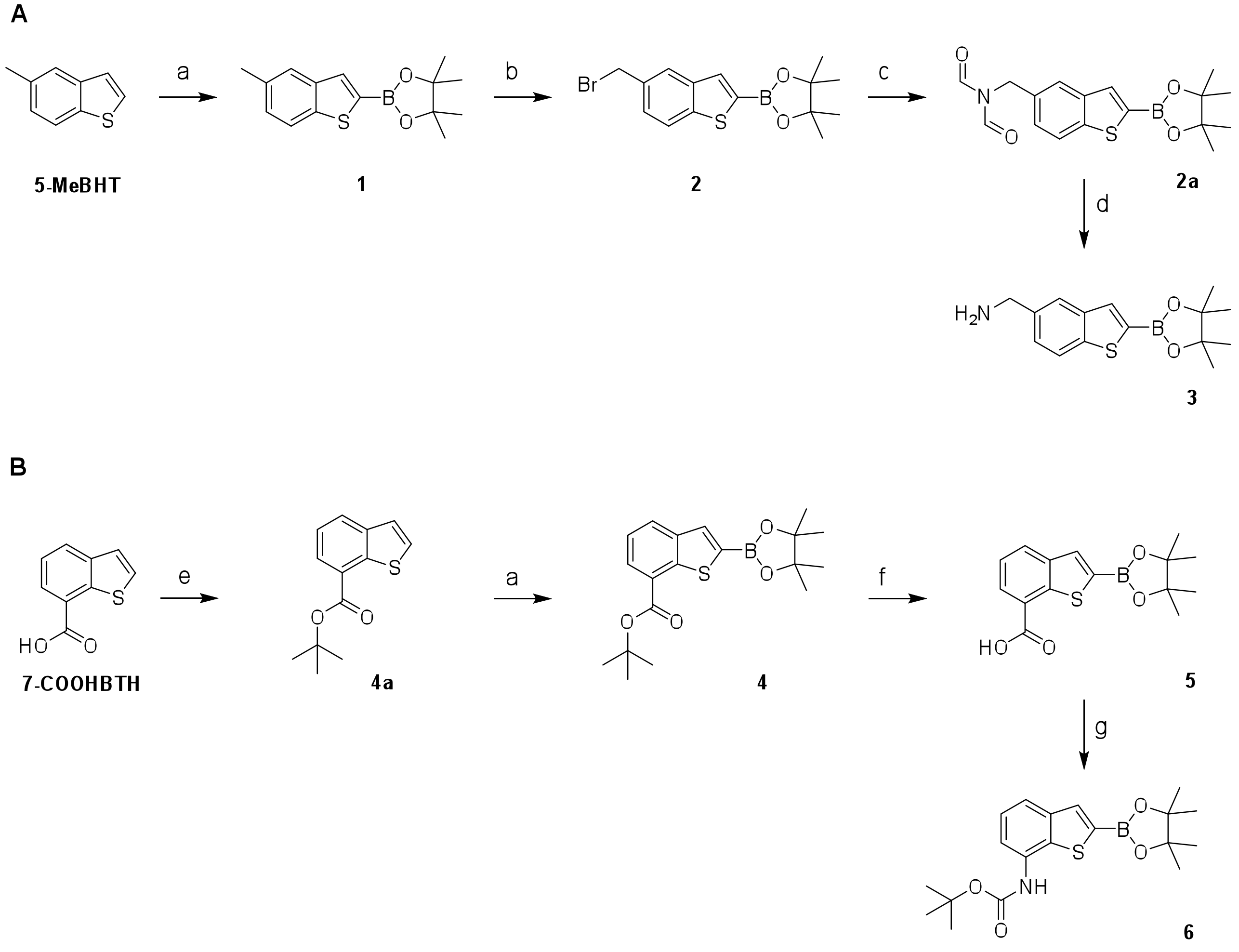

Synthesis scheme. A. C5 position derivatization: synthesis of 5 benzo[b]thiophen-2-ylboronic acid derivatives. B. C7 position derivatization: synthesis of 7 benzo[b]thiophen-2-ylboronic acid derivatives; (a) 4,4,5,5-tetramethyl-1,3,2-dioxaborolane, 4,4′-di-tert-butyl-2,2′-dipyridyl, (1,5-cyclooctadiene) (methoxy)iridium(I) dimer, n-hexane dry, room temperature (RT); (b) NBS, benzoyl peroxide, CCl4, 80 °C; (c) sodium diformylamide, CH3CN, reflux; (d) 5% ethanolic HCl, reflux; (e) perchloric acid, tertbutyl acetate, RT; (f) trifluoroacetic acid, dichloromethane (DCM), RT; (g) diphenyl phosphoryl azide, triethylamine (TEA), tert-butanol, toluene, 100° C.

Chemical synthesis

Materials. 5-Methylbenzo[b]thiophene (5-MeTHB), benzo[*b*]thiophene-7-carboxylic acid (7-COOHTHB) and all reagents were purchased from Aldrich, Sigma and Fluka and were of reagent grade. Reaction progress was monitored by TLC on pre-coated silica gel 60 F_{254} plates (Merck). Silica gel (60M; 230-400 mesh, ASTM) was used for column chromatography. The purity of all synthesized compounds was determined by elemental analyses, performed on a Perkin-Elmer 240C instrument, and all values were within ± 0.4% of the theoretical values. Yields refer to purified products and were not optimized. All compounds were characterized by ^{1}H NMR on AC200 and Bruker MX400 WB instruments (CIGS, University of Modena e Reggio Emilia). Some compounds were also characterized through 2D NMR and ^{13}C NMR. Unless otherwise stated, spectra were recorded in DMSO-d_6 or $CDCl_3$. Chemicals shifts are reported in ppm from tetramethylsilane as an internal standard. LC-MSIT analysis was performed through HPLC Agilent 1200 Ion Trap LC/MS Agilent 6310 device, source of ionization: ESI. Column: ZORBAX SB-C18 2.1X30 mm, particles of 3.5µM. For the boronic derivatives the MS analysis was in a few cases performed without chromatographic column.

Synthetic procedures.

4,4,5,5-tetramethyl-2-(5-methylbenzo[b]thiophen-2-yl)-1,3,2-dioxaborolane (1). Iridium complex [(1,5-cyclooctadiene) (methoxy)iridium(I)dimer] (0.05 mmol) and 4,4'-di-*tert*-butyl-2,2'dipyridyl (0,1 mmol) were flushed under nitrogen in a two neck flask. Anhydrous n-hexane (5 mL), 4,4,5,5-tetramethyl-1,3,2-dioxaborolane (4.5 mmol) and 5-methylbenzo[*b*]thiophene (3 mmol) were added to reaction. The mixture was stirred at room temperature under nitrogen for 4 hours, then it was treated with water (10 mL) and extracted with dichloromethane (3x10 mL). The organic layer was dried with Na_2SO_4 and concentrated in vacuum affording **1** as brown oil. Yield: 95%

^{1}H NMR (400 MHz, DMSO): δ 1.42 (s, 12H, H-11), 2.53 (s, 3H, H-9), 7.25(d, *J=8.2 Hz*, 1H, H-6) 7.68 (s, 1H, H-4), 7.82 (d, *J=8.2 Hz*, 1H, H-7), 7.84 (s, 1H, H-2); ^{13}C NMR (100 MHz, DMSO): δ

21.4 (C-9), 24.8 (C-11), 84.4 (C-10), 122.1 (C-7), 124.1 (C-4), 127.3 (C-6), 133.8 (C-2), 134.1 (C-5), 140.9 (C-8), 141.0 (C-3), (quaternary CB not seen); analysis (calcd., found for $C_{15}H_{19}BO_2S$): C (65.71, 65.74), H (6.98, 6.96).

2-(5-(bromomethyl)benzo[b]thiophen-2-yl)-4,4,5,5-tetramethyl-1,3,2-dioxaborolane (2). N-bromosuccinimide (6 mmol) was added to 6 mmol of **1** in CCl_4 (40 mL). The mixture was heated under reflux and irradiated with UV lamp, and then benzoyl peroxide (0.58 mmol) was added. The mixture was cooled with ice and the resulting precipitate, constituted by succinimide, was removed by filtration. Filtrate was concentrated under vacuum and the crude residue was crystallized several times from hexane, affording **2** as violet solid. Yield: 62%; mp 87-90 °C. ^{1}H NMR (400 MHz, DMSO): δ 1.39 (s, 12H, H-11), 4.65 (s, 2H, H-9), 7.42 (dd, 1H, H-6), 7.88 (m, 3H, H-2, H-4, H-7); ^{13}C NMR (100 MHz, DMSO): δ 24.7 (C-11), 30.8 (C-9), 85.4 (C-10), 121.9 (C-7), 124.7 (C-4), 126.9 (C-2), 137.2 (C-6), 137.6 (C-5), 140.8 (C-8), 141.1 (C-3).(quaternary CB not seen); analysis (calcd., found for $C_{15}H_{18}BBrO_2S$): C (51.03, 51.11), H (5.14, 5.19).

N-formyl-N-((2-(4,4,5,5-tetramethyl-1,3,2-dioxaborolan-2-yl)benzo[b]thiophen-5-yl)methyl)formamide (2a). Sodium diformylamide (0.50 mmol) was added to a solution of **2** (0.42 mmol) in CH_3CN (10 mL) and the mixture was heated for 7 h under reflux. The cooled mixture was filtered and washed with CH_3CN. The combined filtrate was concentrated under reduced pressure. Crystallization of the crude product from CH_2Cl_2/pentane afforded **2a**. Yield: 75%; mp 125-128 °C. ^{1}H NMR (400 MHz, DMSO): δ 1.38 (s, 12H, H-11), 4.91 (s, 2H, H-9), 7.41 (dd, 1H, H-6), 7.88 (m, 3H, H-2, H-4, H-7), 8.91 (br s, 2H, H-12); ^{13}C NMR (100 MHz, $CDCl_3$): δ 21.9 (C-9), 25.7 (C-11), 85.0 (C-10), 122.8 (C-7), 124.8 (C-6), 128 (C-2), 134.4 (C-4), 134.8 (C-3), 141.6 (C-8), 141.8 (C-5), C-1 and C-12 were not seen; analysis (calcd., found for $C_{17}H_{20}BNO_4S$): C (59.15, 59.23), H (5.84, 5.88), N (4.06, 4.17).

(2-(4,4,5,5-tetramethyl-1,3,2-dioxaborolan-2-yl)benzo[b]thiophen-5-yl)methanamine (3). A mixture of **2a** (0.3 mmol) and 5% ethanolic HCl (2 mL, freshly prepared from 37% HCl and EtOH) was refluxed for 3 h and then evaporated under reduced pressure to dryness. The crude product was washed with Et_2O, affording **3** as crystalline solid. Yield: 77%; mp >220 °C. ^{1}H NMR (400 MHz, DMSO): δ 1.31 (s, 12H, H-11), 4.15 (s, 2H, H-9), 7.55 (dd, 1H, H-6), 7.92 (s, 1H, H-4), 8.03 (s, 1H, H-2), 8.08 (d, 1H, H-7), 8.38 (br s, 2H); ^{13}C NMR (100 MHz, DMSO): *δ* 24.4 (C-11), 42.1 (C-9), 84.3 (C-10), 122.7 (C-7), 124.9 (C-6), 126.4(C-2), 130.2 (C-4), 134.2 (C-3), 139.9 (C-8), 142.6 (C-5), (quaternary CB not seen). Found ESI-MS m/z: 290.14 [M+1]; analysis (calcd., found for $C_{15}H_{20}BNO_2S$): C (62.30, 62.39), H (6.97, 7.02), N (4.84, 4.86).

***tert*-butyl benzo[b]thiophene-7-carboxylate (4a).** Benzo[b]thiophene-7-carboxylic acid (2.77 mmol) was suspended in 10 mL of tert-butyl acetate, then perchloric acid (0.92 mmol) was added dropwise. The mixture was stirred for 24 h at room temperature, then it was treated with NaHCO3 until basic pH, diluted with dichloromethane, washed with brine (3 x 10 mL), dried and concentrated *in vacuum*. The product was purified through chromatography (eluent: 8 cyclohexane\2 ethyl acetate). Yield 45%. ^{1}H NMR (400 MHz, DMSO): *δ* 1.73 (s, 9H, H-11), 7.43 (d, 1H, J=5.5 Hz, H-2), 7.48 (t, 1H, J=7.6 Hz, H-5), 7.60 (d, 1H, J=5.6 Hz, H-1), 8.03 (dd, J=7.8 Hz, 1.3 Hz, 1H, H-4), 8.13 (dd, J=7.5 Hz, 1.3 Hz, 1H, H-6); ^{13}C NMR (100 MHz, DMSO): δ 28.3 (C-11), 82.1 (C-10), 122.7 (C-1), 123.5 (C-5), 126.0 (C-7), 126.8 (C-6), 127.7 (C-4), 128.7 (C-2), 139.5 (C-8), 140.9 (C-3) 165.3 (C-9); analysis (calcd., found for $C_{13}H_{14}O_2S$): C (66.64, 66.69), H (6.02, 6.06).

2-(4,4,5,5-Tetramethyl-[1,3,2]dioxaborolan-2-yl)-benzo[b]thiophene-7-carboxylic acid tert-butyl ester (4). Iridium complex [(1,5-cyclooctadiene)(methoxy)iridium (I) dimer] (0,02 mmol) and 4,4'-di-*tert*-butyl-2,2'dipyridyl (0,04 mmol) were flushed with nitrogen in a two neck flask.

anhydrous n-hexane (5 mL), pinacol borane (1.8 mmol) and **4a** (1.2 mmol) were added to reaction. The mixture was stirred at RT under nitrogen for 4 hours. The mixture was treated with water and extracted with DCM. The organic layer was dried over Na_2SO_4 and concentrated *in vacuum* affording **4** as brown oil. Yield 95%. ^{1}H NMR (400 MHz, DMSO): δ 1.41 (s, 12H, H-13), 1.72 (s, 9H, H-11), 7.46 (t, 1H, J=7.6 Hz, H-5), 7.95 (s, 1H, H-2), 8.05 (dd, J= 7.9 Hz, 1.3 Hz, 1H, H-4), 8.14 (dd, J=7.4 Hz, 1.1 Hz, 1H, H-6); ^{13}C NMR (100 MHz, DMSO): δ 24.8 (C-13), 28.3 (C-11), 82.1 (C-10), 84.5 (C-12), 123.6 (C-5), 126.2 (C-7), 127.7 (C-6), 128.6 (C-4), 133.4 (C-2), 141.6 (C-3), 143.5 (C-8), 165.2 (C-9); analysis (calcd., found for $C_{19}H_{25}BO_4S$): C (63.34, 63.42), H (6.99, 7.04).

2-(4,4,5,5-Tetramethyl-[1,3,2]dioxaborolan-2-yl)-benzo[b]thiophene-7-carboxylic acid (5). Compound **4** was treated with a solution of 50% trifluoroacetic acid in dichloromethane for 5 hours at RT, then it was concentrated *in vacuum*. Yield: 75%.

^{1}H NMR (400 MHz, $CDCl_3$): δ 1.34 (s, 12H, H-11), 7.55 (t, 1H, *J=7.6 Hz,* H-5), 7.98 (s, 1H, H-2), 8.10 (dd, *J= 7.4 Hz, 1.1 Hz*, 1H, H-4), 8.21 (dd, *J=7.8 Hz, 1.1 Hz*, 1H, H-6); ^{13}C NMR (100 MHz, $CDCl_3$): δ 21.1 (C-11), 122.1 (C-7), 123.3 (C-1), 123.4 (C-4), 125.9 (C-6), 126.2 (C-2), 133.8 (C-5), 137.0 (C-8), 139.9 (C-3). (C-1 and C-9 were not seen). Found ESI-MS m/z: 303.09 [M-1]; analysis (calcd., found for $C_{15}H_{17}BO_4S$): C (59.23, 59.25), H (5.63, 5.68).

(7-((*tert*-butoxycarbonyl)amino)benzo[b]thiophen-2-yl)boronic acid (6). Compound **5** (0.6 mmol) was suspended in 6 mL of toluene and 1 mL of tert-butanol, diphenylphosphorilazide (0.9 mmol) and triethylamine (1.5 mmol) were added. The mixture was stirred for 3 hours at RT, then 1 ml of *tert*-butanol was added and warmed to 100°C for 18 hours. The mixture was quenched with water and extracted with DCM (3x5 mL). The organic layers were combined, washed with water (3 x 5mL), dried and concentrated *in vacuo*. The product was purified through RP-Chromatography.

The final product was recovered both in the pinacol-protected form and as free boronic acid. Yield: 40%. ^{1}H NMR (400 MHz, DMSO): δ 1.56 (s, 9H, H-11), 7.34 (t, 1H, *J=7.7 Hz*, H-5), 7.46 (d, *J= 7.5 Hz*, 1H, H-6), 7.68 (d, *J=7.8 Hz*, 1H, H-4), 7.88 (s, 1H, H-2); ^{13}C NMR (100 MHz, DMSO): δ 27.4 (C-11), 79.5 (C-10), 119.1 (C-6), 120.6 (C-4), 124.3 (C-5), 132.5 (C-7), 133.0 (C-2), 137.2 (C-8), 141.9 (C-3). C-1 and C-9 were not seen; analysis (calcd., found for $C_{13}H_{16}BNO_4S$): C (53.26, 53.21), H (5.50, 5.54), N (4.78, 4.75).

Scheme S2.

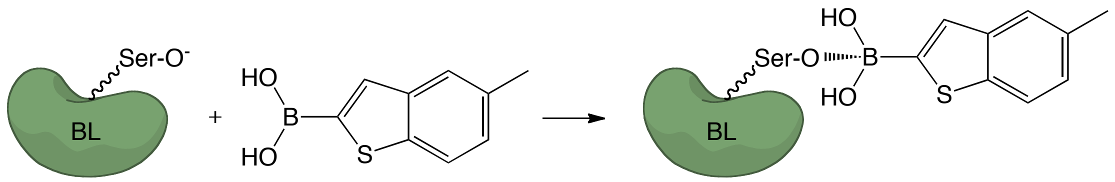

Mechanism of action of a boronic-based inhibitor, *i.e*. compound **2**. The configurational change of the boronic group is highlighted.

Figure S2.

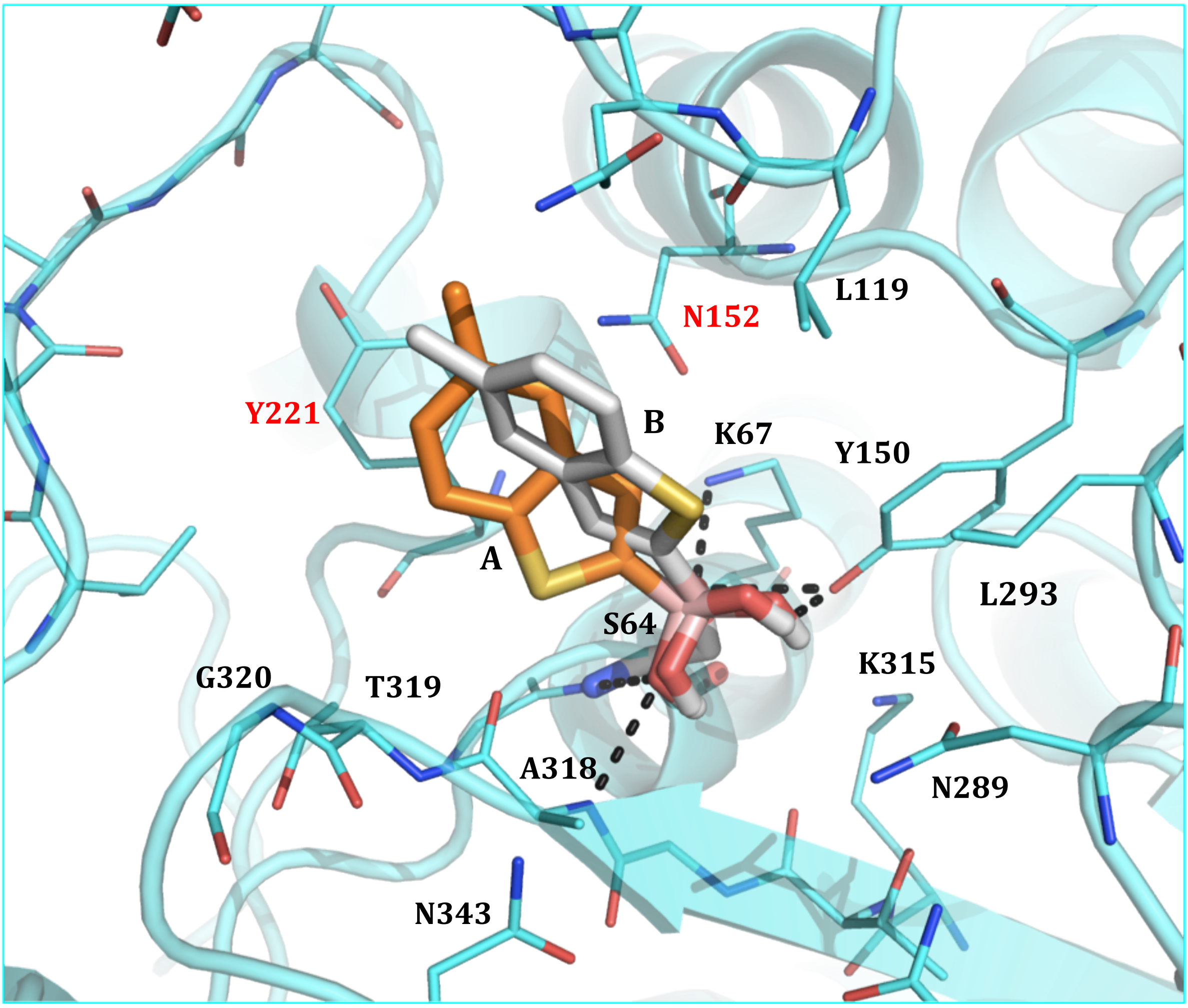

Possible orientation assumed by compound 2 in AmpC binding site. A. pi-pi interaction are formed by the ligand benzene ring with Tyr210 (orange coloured ligand). B. dipole-quadrupole interactions are formed by the ligand benzene ring with Asn152 side-chain (yellow coloured ligand). Crucial residues are red labelled. Hydrogen bonds are represented by black dashed lines.

References

1 Lahiri, S. D., Johnstone, M. R., Ross, P. L., McLaughlin, R. E., Olivier, N. B. & Alm, R. A. Avibactam and class C beta-lactamases: mechanism of inhibition, conservation of the binding pocket, and implications for resistance. *Antimicrob. Agents Chemother.* **58**, 5704-5713, (2014).

2 Lahiri, S. D., Mangani, S., Jahic, H., Benvenuti, M., Durand-Reville, T. F., De Luca, F., Ehmann, D. E., Rossolini, G. M., Alm, R. A. & Docquier, J. D. Molecular basis of selective inhibition and slow reversibility of avibactam against class D carbapenemases: a structure-guided study of OXA-24 and OXA-48. *ACS Chem Biol* **10**, 591-600, (2015).

3 Docquier, J. D., Lamotte-Brasseur, J., Galleni, M., Amicosante, G., Frere, J. M. & Rossolini, G. M. On functional and structural heterogeneity of VIM-type metallo-beta-lactamases. *J Antimicrob Chemother* **51**, 257-266, (2003).

4 Quero, G., Zuppolini, S., Consales, M., Diodato, L., Vaiano, O., Venturelli, A., Santucci, M., Spyrakis, F., Costi, M., Giordano, A., Borriello, A., Cutolo, A. & Cusano, A. Long period fiber grating working in reflection mode as valuable biosensing platform for the detection of drug resistant bacteria. *Sensors and Actuators B: Chemical* **230**, 510-520, (2016).

5 Lahiri, S. D., Mangani, S., Durand-Reville, T., Benvenuti, M., De Luca, F., Sanyal, G. & Docquier, J. D. Structural insight into potent broad-spectrum inhibition with reversible recyclization mechanism: avibactam in complex with CTX-M-15 and Pseudomonas aeruginosa AmpC beta-lactamases. *Antimicrob. Agents Chemother.* **57**, 2496-2505, (2013).

6 Ourghanlian, C., Soroka, D. & Arthur, M. Inhibition by Avibactam and Clavulanate of the beta-Lactamases KPC-2 and CTX-M-15 Harboring the Substitution N132G in the Conserved SDN Motif. *Antimicrob Agents Chemother* **61**, (2017).

7 Santillana, E., Beceiro, A., Bou, G. & Romero, A. Crystal structure of the carbapenemase OXA-24 reveals insights into the mechanism of carbapenem hydrolysis. *Proc Natl Acad Sci U S A* **104**, 5354-5359, (2007).

8 Borgianni, L., Vandenameele, J., Matagne, A., Bini, L., Bonomo, R. A., Frere, J. M., Rossolini, G. M. & Docquier, J. D. Mutational analysis of VIM-2 reveals an essential determinant for metallo-beta-lactamase stability and folding. *Antimicrob Agents Chemother* **54**, 3197-3204, (2010).

9 Liang, Z., Li, L., Wang, Y., Chen, L., Kong, X., Hong, Y., Lan, L., Zheng, M., Guang-Yang, C., Liu, H., Shen, X., Luo, C., Li, K. K., Chen, K. & Jiang, H. Molecular basis of NDM-1, a new antibiotic resistance determinant. *PLoS One* **6**, e23606, (2011).
